# Comparison of in-person versus tele-ultrasound point-of-care ultrasound training during the COVID-19 pandemic

**DOI:** 10.1186/s13089-021-00242-6

**Published:** 2021-09-06

**Authors:** Nilam J. Soni, Jeremy S. Boyd, Gregory Mints, Kevin C. Proud, Trevor P. Jensen, Gigi Liu, Benji K. Mathews, Christopher K. Schott, Linda Kurian, Charles M. LoPresti, Phil Andrus, Robert Nathanson, Natalie Smith, Elizabeth K. Haro, Michael J. Mader, Jacqueline Pugh, Marcos I. Restrepo, Brian P. Lucas

**Affiliations:** 1 South Texas Veterans Health Care System, San Antonio, TX USA; 2 Department of Medicine, UT Health San Antonio, San Antonio, TX USA; 3 Department of Emergency Medicine, Veterans Affairs - Tennessee Valley Healthcare System, Nashville, TN USA; 4 Department of Emergency Medicine, Vanderbilt University Medical Center, Nashville, TN USA; 5 Division of Hospital Medicine, Weill Cornell Medicine, New York, NY USA; 6 Division of Hospital Medicine, University of California San Francisco Medical Center at Parnassus, San Francisco, CA USA; 7 Division of General Internal Medicine, Department of Medicine, Johns Hopkins School of Medicine, Baltimore, MD USA; 8 Department of Hospital Medicine, Regions Hospital, HealthPartners, St. Paul, MN USA; 9 Department of Critical Care Medicine, Veterans Affairs of Pittsburgh Health Care System, Pittsburgh, PA USA; 10 Departments of Critical Care Medicine and Emergency Medicine, University of Pittsburgh Medical Center, University of Pittsburgh, Pittsburgh, PA USA; 11 Division of Hospital Medicine, Donald and Barbara Zucker School of Medicine at Hofstra/Northwell, Hempstead, NY USA; 12 Louis Stokes Cleveland Veterans Affairs Medical Center, Cleveland, OH USA; 13 Case Western Reserve University School of Medicine, Cleveland, OH USA; 14 Department of Emergency Medicine, Donald and Barbara Zucker School of Medicine at Hofstra/Northwell, Hempstead, NY USA; 15 White River Junction VA Medical Center, White River Junction, VT USA; 16 Department of Medicine, Dartmouth Geisel School of Medicine, Hanover, NH USA; 17 University of Texas Health San Antonio, South Texas Veterans Health Care System, 7703 Floyd Curl Drive, MC 7982, San Antonio, TX 78229 USA

## Abstract

**Background:**

Lack of training is currently the most common barrier to implementation of point-of-care ultrasound (POCUS) use in clinical practice, and in-person POCUS continuing medical education (CME) courses have been paramount in improving this training gap. Due to travel restrictions and physical distancing requirements during the COVID-19 pandemic, most in-person POCUS training courses were cancelled. Though tele-ultrasound technology has existed for several years, use of tele-ultrasound technology to deliver hands-on training during a POCUS CME course has not been previously described.

**Methods:**

We conducted a retrospective observational study comparing educational outcomes, course evaluations, and learner and faculty feedback from in-person versus tele-ultrasound POCUS courses. The same POCUS educational curriculum was delivered to learners by the two course formats. Data from the most recent pre-pandemic in-person course were compared to tele-ultrasound courses during the COVID-19 pandemic.

**Results:**

Pre- and post-course knowledge test scores of learners from the in-person (*n* = 88) and tele-ultrasound course (*n* = 52) were compared. Though mean pre-course knowledge test scores were higher among learners of the tele-ultrasound versus in-person course (78% vs. 71%; *p* = 0.001), there was no significant difference in the post-course test scores between learners of the two course formats (89% vs. 87%; *p* = 0.069). Both learners and faculty rated the tele-ultrasound course highly (4.6–5.0 on a 5-point scale) for effectiveness of virtual lectures, tele-ultrasound hands-on scanning sessions, and course administration. Faculty generally expressed less satisfaction with their ability to engage with learners, troubleshoot image acquisition, and provide feedback during the tele-ultrasound course but felt learners completed the tele-ultrasound course with a better basic POCUS skillset.

**Conclusions:**

Compared to a traditional in-person course, tele-ultrasound POCUS CME courses appeared to be as effective for improving POCUS knowledge post-course and fulfilling learning objectives. Our findings can serve as a roadmap for educators seeking guidance on development of a tele-ultrasound POCUS training course whose demand will likely persist beyond the COVID-19 pandemic.

**Supplementary Information:**

The online version contains supplementary material available at 10.1186/s13089-021-00242-6.

## Background

Competency in point-of-care ultrasound (POCUS) requires basic knowledge of ultrasound technology, skills in image acquisition and interpretation, and ability to integrate findings into clinical decision-making. Historically, practicing clinicians have gained POCUS skills by attending in-person continuing medical education (CME) courses, and these courses have been shown to be effective [1].

Due to travel restrictions and physical distancing requirements during the COVID-19 pandemic, most in-person POCUS training courses were cancelled, although the demand for POCUS training may have increased given the utility of POCUS in COVID-19 [2]. Many traditional lecture-based CME courses were converted to virtual courses relatively easily during the pandemic, but a major challenge in creating a virtual POCUS CME course is the need for hands-on training with live and simulation models, which is the primary reason many learners attend an in-person POCUS course. Tele-ultrasound technology has been used to perform remote scanning of patients [3, 4], especially in resource-limited settings [5, 6], but few studies have described its use for virtual hands-on training of clinicians [7–11]. Furthermore, no prior studies have described use of tele-ultrasound technology for hands-on POCUS training as part of an accredited CME course for practicing clinicians.

We converted an established in-person POCUS CME course to a tele-ultrasound course during the COVID-19 pandemic. The goal of the tele-ultrasound POCUS CME course was to provide the same educational experience, including hands-on training, using a virtual format. We compared the change in POCUS knowledge of learners, fulfillment of course objectives, and faculty and learner feedback of the in-person versus tele-ultrasound POCUS CME courses.

## Methods

We conducted a retrospective observational study comparing an established in-person POCUS course in February 2020 (pre-pandemic) versus new tele-ultrasound POCUS courses in July 2020 and February 2021 during the COVID-19 pandemic. The same POCUS educational curriculum was used for the in-person and tele-ultrasound courses, although the format of delivering educational content differed. All learners and faculty were considered for inclusion and only excluded if their pre- or post-course datasets were incomplete. The University of Texas Health San Antonio Investigational Review Board deemed this retrospective educational study to be exempt from formal review.

### In-person course

We held in-person POCUS courses every 6–12 months from 2013 until the COVID-19 pandemic in early 2020. These 2-day courses combined focused lectures, image review sessions, and hands-on scanning sessions (Additional file 1: Table S1). Both live models and simulators were used. A learner-to-faculty ratio of 3:1 or 2:1 was maintained for all hands-on training. Data from the most recent in-person course in February 2020 were used for comparison to the tele-ultrasound courses.

### Tele-ultrasound course

To meet the ongoing demand of POCUS training during the COVID-19 pandemic, we converted our traditional 2-day in-person course into a 4-week tele-ultrasound course. While the number of CME hours from didactics and hands-on training stayed the same (16.25 h) (Additional file 1: Table S2), the curriculum was spread over 4 weeks for two reasons. First, learners and faculty needed additional time to become comfortable using the tele-ultrasound software. Second, our course coordinators were online to connect faculty and learners, initiate the hands-on scanning sessions, and troubleshoot any problems that arose during the session. Thus, we scheduled individual hands-on scanning sessions at different times, rather than all learners and faculty scanning simultaneously. The learner-to-faculty ratio was 2:1 or 1:1 for all hands-on scanning sessions. Hands-on scanning objectives of the in-person and tele-ultrasound courses were similar (Additional file 1: Tables S3 and S4).

**Fig. 1:**
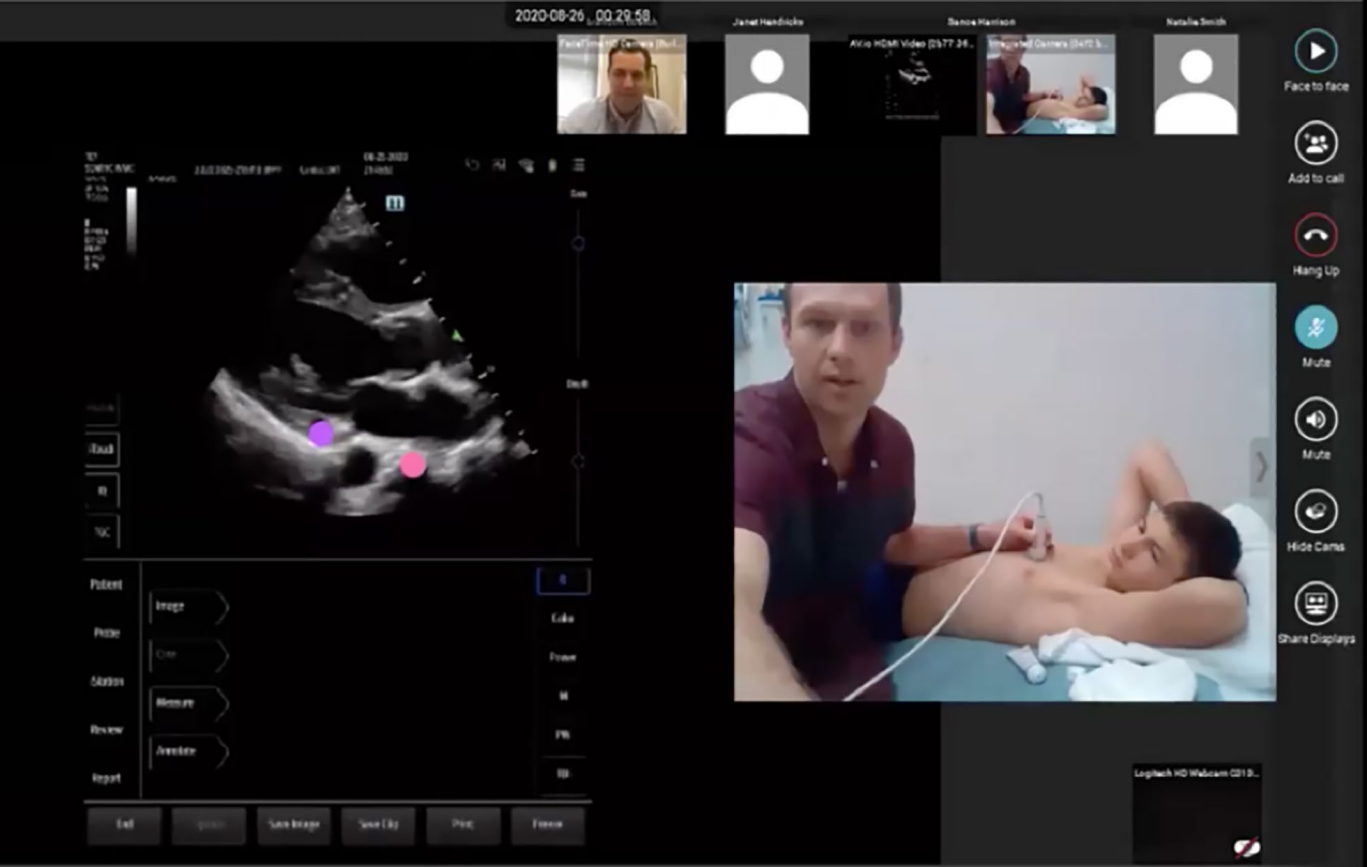
Tele-ultrasound hands-on scanning session. Live hands-on scanning sessions were conducted using tele-ultrasound software (Reacts by Philips & Innovative Imaging Technologies). The platform allowed simultaneous visualization of the learner’s hands position (right) and ultrasound screen (left) which allowed faculty to guide learners in image acquisition. Both faculty and learners could point to structures on the ultrasound screen using different colored pointers (left)

We utilized tele-ultrasound software (Reacts by Philips & Innovative Imaging Technologies, Montreal, Canada). This software allowed simultaneous visualization of a learner’s hand position and ultrasound screen (Fig. 1).

We piloted our tele-ultrasound course with 12 learners at four remote critical access hospitals in southeastern Alaska in July 2020, followed by a larger course with 43 learners from 14 different states in the United States and abroad in February 2021. Twenty-six experienced POCUS faculty specializing in internal medicine, hospital medicine, critical care, and emergency medicine from 15 different U.S. institutions provided virtual hands-on POCUS instruction by tele-ultrasound. Faculty and learners were scheduled for 2-h hands-on scanning sessions that allowed for 60–90 min of actual scanning. Live models were relatives of learners, course participants, or volunteer staff or patients.

Learners completed pre- and post-course knowledge tests (Additional file 1: File S1), CME course evaluations (Additional file 1: File S2), tele-ultrasound course evaluations (Additional file 1: File S3), and participated in a live post-course debriefing. The knowledge test included 30 questions focusing on fundamentals of ultrasonography, image interpretation, and integration of POCUS findings in clinical decision-making. After both the in-person and tele-ultrasound POCUS courses, learners completed a standard CME course evaluation that gathered both quantitative and qualitative feedback. Learners evaluated fulfillment of course objectives and quality of lectures and hands-on scanning sessions by rating their agreement with standardized statements on a scale of 1–5 (1 = “not at all” or “low agreement”; 5 = “excellent” or “high agreement”). We gathered qualitative feedback from learners of the in-person and tele-ultrasound courses in three domains: (1) teaching effectiveness of faculty for hands-on sessions; (2) new knowledge or practice strategies gained; and (3) practice changes implemented after attending the course. Qualitative data were collected from learners using free text boxes in the CME course evaluation and tele-ultrasound course evaluation, and during live post-course debriefing sessions. Faculty evaluated the tele-ultrasound course by completing the same tele-ultrasound course evaluation as learners that included faculty-specific questions and during a live post-course debriefing session for faculty.

Mean knowledge test scores and differences in scores were compared using the Mann–Whitney test. Paired *t*-tests were used to test the hypothesis of an increase in knowledge test scores from pre- to post-course for each course format. Proportions of learners rating “excellent” on course evaluations were compared for each objective using the *Z*-test for proportions. A *p*-value of < 0.05 was considered statistically significant. Qualitative data from learners and faculty were reviewed and discussed by two co-authors (NJS, EKH) and recurring themes were compiled.

## Results

Eighty-eight learners from the in-person course and 52 from the tele-ultrasound course completed all pre- and post-course assessments. The vast majority of learners were novices with no or minimal experience using POCUS and did not or infrequently used POCUS (few times per year). Twenty-six faculty participated in the tele-ultrasound course, and 54% of the same faculty taught both the in-person and tele-ultrasound courses. Learner and faculty characteristics are displayed in Additional file 1: Table S5.

The mean pre-course knowledge test scores were higher for learners in the tele-ultrasound versus in-person course (78% vs. 71%; *p* = 0.001), but the post-course scores of learners in the tele-ultrasound course improved to a similar level as those in-person course (89% vs. 87%; *p* = 0.069), although with a lower net overall improvement. Thus, learners demonstrated a significant improvement in POCUS knowledge after participating in either the in-person or tele-ultrasound course. Further, both courses fulfilled course objectives similarly based on CME course evaluations (Table 1).

**Table 1:** Comparison of knowledge test scores and fulfillment of course objectives by the in-person vs. tele-ultrasound POCUS courses

	In-person (n = 88)	Tele-ultrasound (n = 52)	p-value
Knowledge test scores			
Pre-course (%)			
Mean	71	78	0.001
Median [IQR]	73 [60–83]	83 [71–85]	
Range	34–98	49–95	
Post-course (%)			
Mean	87	89	0.069
Median [IQR]	88 [83–91]	90 [85–93]	
Range	49–98	54–100	
Differences (%)			
Mean	16	11	0.017
Median [IQR]	12 [7–22]	7 [2–18]	
Range	(− 7)–51	(− 12)–39	
*p*-value	< 0.0001	< 0.0001	
Fulfillment of course objectives^a^			
Fundamental principles of ultrasound technology and basic operation of a portable ultrasound machine	4.7	4.7	0.81
Techniques to perform focused diagnostic ultrasound examinations at the bedside, including imaging of the heart, lungs, abdomen, and lower extremity veins	4.8	4.8	0.53
Techniques to guide performance of bedside procedures with ultrasound guidance, including central venous catheterization, thoracentesis, paracentesis, and lumbar puncture	4.5	4.4	0.32
Indications, basic protocols, and limitations of bedside ultrasound imaging	4.7	4.6	0.65
Practice interpretation of normal and abnormal ultrasound images	4.7	4.7	0.81
Mentored scanning with experienced faculty to learn hands-on imaging techniques	4.7	4.7	0.70

^a^Mean scores per 5-point scale (1 = not at all; 2 = minimal; 3 = neutral; 4 = good; 5 = excellent) with *p*-values calculated for proportion giving an excellent rating

Both learners and faculty of the tele-ultrasound POCUS course completed a separate course evaluation assessing the tele-ultrasound course format (Additional file 1: Tables S6 and S7). Overall, learners rated the tele-ultrasound course highly (4.6–5.0 on a 5-point scale) for lectures, tele-ultrasound hands-on scanning sessions, and course administration.

Similar to learners, the majority of faculty rated the effectiveness of the tele-ultrasound course administration and hands-on sessions highly; however, the virtual lectures were felt to be less effective by faculty than learners (3.7 vs. 4.8). Compared to learners, faculty generally expressed less satisfaction with the tele-ultrasound scanning sessions regarding their ability to engage learners, troubleshoot image acquisition, and provide feedback. Faculty did not express differences in difficulty of teaching specific cardiac or non-cardiac applications using tele-ultrasound, but difficulty visualizing the learner’s probe position was a common complaint.

We highlight some of the key points gathered from the qualitative feedback. First, regarding teaching effectiveness of faculty during hands-on sessions, some learners of the in-person course disliked faculty taking control of the ultrasound transducer without letting the learner troubleshoot his/her own image. Second, learners in both the in-person and tele-ultrasound courses reported increased confidence in their ability to utilize POCUS in patient care post-course. Third, similar practice changes were anticipated by learners after participating in either the in-person or tele-ultrasound course, the most common being increased frequency of POCUS use in patient care. Additional advantages and disadvantages of the tele-ultrasound course from the perspectives of learners and faculty are presented in Table 2.

**Table 2:** Summary of learner and faculty feedback of the tele-ultrasound courses

	Learners	Faculty
**Lectures**		
Advantages	More convenient and comfortable Recorded lectures good for review Small group size is less intimidating	Flexible scheduling
Disadvantages	Easier to get distracted	Difficult to engage learners
**Hands-on scanning**		
Advantages	Time for self-directed practice in between scanning sessions Personalized teaching Opportunity to troubleshoot scanning without faculty intervening physically Flexible scheduling of sessions	More satisfying teaching 1–2 learners Less exhausting physically Time to convey learner needs between faculty Flexible scheduling of sessions
Disadvantages	Reliable internet signal required Tele-ultrasound software can glitch Learners could benefit from peer observation	Learner fatigue during 1–2 h hands-on sessions Inability to demonstrate skills Struggling learners difficult to guide No control over learners’ setting (lighting, room and model positioning, space limitations, etc.)
**Procedure training**		
Advantages	None	None
Disadvantages	Limited number of simulation models can be sent to learner	Inability to demonstrate technique on simulation model
**General**		
Advantages	No travel required Lower total training costs	Lower risk of COVID-19 exposure Faculty honoraria equitably paid based on involvement in course
Disadvantages	Multi-week course is time consuming Time zones differences can cause scheduling errors	Strong administrative support is imperative for course coordination Extended course duration compared to 2-day in-person course

## Discussion

For many learners, the primary reason to attend an in-person POCUS CME course is the opportunity to practice hands-on scanning on live models and ultrasound-guided procedures on simulators under the guidance of expert faculty. We have demonstrated that hands-on scanning is feasible using tele-ultrasound technology, and a tele-ultrasound POCUS CME course can improve learners’ post-course knowledge to a similar level as an in-person course. We have also identified important advantages and disadvantages of the two course formats and considerations for course directors seeking to develop a tele-ultrasound POCUS CME course.

Learners’ POCUS knowledge improved significantly after participating in either the in-person or tele-ultrasound POCUS course. This finding has important implications for future dissemination of POCUS training since lack of training is the number one barrier to POCUS implementation [12–14]. Historically, POCUS training has been obtained primarily through in-person courses, but the limited availability and required travel of in-person courses are important limitations. Avoiding travel, reducing time away from work and personal responsibilities, and reducing total training costs were unique advantages of the tele-ultrasound course format.

Additional advantages and disadvantages of a tele-ultrasound POCUS course surfaced from qualitative feedback gathered. Most important, faculty felt learners completed the tele-ultrasound course with a better basic POCUS skillset than the traditional in-person course, which may be attributed to the longer course duration (4 weeks vs. 2 days), lower learner-to-faculty ratio (1 or 2:1 vs. 2 or 3:1), and greater comfort and convenience for learners scanning in their home environment. Learners commented that the multi-week format of the tele-ultrasound courses allowed them more time to absorb the course material and practice scanning in between hands-on sessions. Further, the total number of hours spent scanning by faculty was higher with the tele-ultrasound courses because the learner-to-faculty ratios were lower; however, faculty did not lose time traveling as they would for an in-person course which was an important advantage to some faculty. Finally, learners preferred to troubleshoot their own images without faculty intervening physically by taking control of the transducer. This was an advantage per learners, but a disadvantage per faculty who often preferred to take physical control of the transducer to intervene or demonstrate image acquisition.

For course directors interested in developing a tele-ultrasound POCUS course, we highlight three important considerations: selection of videoconferencing and tele-ultrasound software, selection of learners and faculty, and course coordination.

First, several general videoconferencing platforms exist (e.g., Zoom, Skype, Cisco WebEx, GoToMeeting, Microsoft Teams, BlueJeans Meetings, Google Hangouts Meet, and Jabber). Essential features for a virtual POCUS course include high-quality transmission of ultrasound images without a lag or choppiness, sufficient online storage for recording lectures, and ease of use for both learners and faculty with features (chat, screensharing, etc.). Regarding tele-ultrasound platforms, both ultrasound-specific and general videoconferencing software can be used. Two examples of ultrasound-specific software are Reacts (Philips/Innovative Imaging Technologies, Montreal, Canada) and Butterfly Teleguidance (Butterfly Network, Guilford, CT, USA). A unique advantage of these tele-ultrasound platforms is simultaneous camera and ultrasound image transmission using a cellular or internet signal, allowing faculty to coach learners in image acquisition while simultaneously seeing the learner’s hand position and ultrasound image. Reacts can be used with most major brands of ultrasound machines, whereas Butterfly Teleguidance is available only with Butterfly ultrasound devices. Some tele-ultrasound software allow faculty to remotely adjust ultrasound machine settings to optimize the image, which can be an important feature if learners are struggling with knobology. General videoconferencing software may be used for tele-ultrasound using remote desktops or screensharing, or by pointing the videoconferencing camera directly on the ultrasound screen, but variable degrees of image degradation occur with the latter option.

Second, success of hands-on scanning sessions using tele-ultrasound is dependent on both the learners and faculty. Learners must have reliable access to an ultrasound device compatible with the selected tele-ultrasound software; a reliable internet signal with sufficient bandwidth to simultaneously transmit ultrasound and video images; and availability of a live model for hands-on scanning. Experienced faculty that have previously demonstrated mastery in guiding learners in image acquisition *without* touching the transducer should be selected for the tele-ultrasound hands-on scanning sessions. All faculty must use standard terminology for transducer manipulation (slide, tilt, rotate, rock) to avoid confusing learners that scan with multiple faculty. Less experienced faculty can learn from more experienced faculty by shadowing during a live tele-ultrasound session.

Third, compared to an in-person course, substantially more educational staff time is needed to coordinate a tele-ultrasound POCUS CME course. Unique tasks included screening and selecting learners and faculty as mentioned above; organizing, packing, and shipping loaned tele-ultrasound equipment to learners; scheduling 2-h tele-ultrasound sessions between learners and faculty; and connecting learners and faculty online during scanning sessions.

Regarding costs, the tele-ultrasound course budget was similar to the in-person course. However, funds were spent differently in the tele-ultrasound course: more funds were spent on educational staff time for course coordination, faculty honoraria for scanning sessions, and purchasing and shipping of tele-ultrasound equipment, while funds were saved on faculty travel and venue expenditures, such as catering and room rental. For learners, there were significant cost savings by avoiding travel.

We recognize our findings have limitations and require confirmation. Though many faculty and learners felt better hands-on scanning skills were achieved by learners upon completion of the tele-ultrasound course, it was not feasible to test learners’ hands-on skills pre- and post-course. Additionally, although both courses had the same number of CME credit hours, the duration of the tele-ultrasound course was 4 weeks while the in-person course was 2 days. Next steps include piloting a 2-day tele-ultrasound course to test the feasibility and logistics of having multiple learners and faculty scanning simultaneously using tele-ultrasound compared to a traditional in-person 2-day course.

In conclusion, we have demonstrated that a virtual POCUS CME course with hands-on scanning sessions using tele-ultrasound is feasible. Both the traditional in-person and tele-ultrasound POCUS courses fulfilled learning objectives and significantly improved learners’ post-course knowledge to a similar level. By improving learners’ access to expert faculty for mentored scanning, tele-ultrasound can help bridge the gap in POCUS training in the coming years.

## Supplementary Information

**Additional file 1**: **Table S1.** Two-day In-person POCUS Course Agenda. **Table S2.** Four-week Tele-ultrasound POCUS Course Agenda. **Table S3.** In-person Course Scanning Session Objectives. **Table S4.** Tele-ultrasound Course Scanning Session Objectives. **File S1.** Pre- and Post-course Knowledge Test. **File S2.** CME Course Evaluation. **File S3.** Tele-ultrasound Course Evaluation. **Table S5.** Characteristics of Learners and Faculty. **Table S6.** Tele-ultrasound Course Evaluations by Learners and Faculty. **Table S7.** Faculty Evaluation of the Tele-ultrasound Course.

## Data Availability

Data are available upon request.

## Abbreviations

CME: Continuing medical education
POCUS: Point-of-care ultrasound

## References

[1] Greenstein YY, Littauer R, Narasimhan M, Mayo PH, Koenig SJ (2016). Effectiveness of a critical care ultrasonography course. Chest.

[2] Mathews BK, Koenig S, Kurian L (2020). Clinical progress note: point-of-care ultrasound applications in COVID-19. J Hosp Med.

[3] Gibson LE, Low SA, Bittner EA, Chang MG (2020). Ultrasound teleguidance to reduce healthcare worker exposure to coronavirus disease 2019. Crit Care Explor.

[4] Olivieri PP, Verceles AC, Hurley JM, Zubrow MT, Jeudy J, McCurdy MT (2020). A pilot study of ultrasonography-naïve operators’ ability to use tele-ultrasonography to assess the heart and lung. J Intensive Care Med.

[5] Britton N, Miller MA, Safadi S, Siegel A, Levine AR, McCurdy MT (2019). Tele-ultrasound in resource-limited settings: a systematic review. Front Public Health.

[6] Rabie NZ, Sandlin AT, Barber KA (2017). Teleultrasound: how accurate are we?. J Ultrasound Med.

[7] Arntfield RT (2015). The utility of remote supervision with feedback as a method to deliver high-volume critical care ultrasound training. J Crit Care.

[8] Schroeder AN, Hall MM, Kruse RC (2020). Sports ultrasound training during a pandemic: developing a “hands-on” skill through distance learning. Am J Phys Med Rehabil.

[9] Dreyfuss A, Martin DA, Farro A (2020). A novel multimodal approach to point-of-care ultrasound education in low-resource settings. West J Emerg Med.

[10] Ramsingh D, Ma M, Le DQ (2019). Feasibility evaluation of commercially available video conferencing devices to technically direct untrained nonmedical personnel to perform a rapid trauma ultrasound examination. Diagnostics.

[11] Salerno A, Tupchong K, Verceles AC, McCurdy MT (2020). Point-of-care teleultrasound: a systematic review. Telemed J E Health.

[12] Wong J, Montague S, Wallace P (2020). Barriers to learning and using point-of-care ultrasound: a survey of practicing internists in six North American institutions. Ultrasound J.

[13] Sanders JL, Noble VE, Raja AS, Sullivan AF, Camargo CA (2015). Access to and use of point-of-care ultrasound in the emergency department. West J Emerg Med.

[14] Stowell JR, Kessler R, Lewiss RE (2017). Critical care ultrasound: a national survey across specialties. J Clin Ultrasound.

