# Supplementary material for: Comparison of in-person versus tele-ultrasound point-of-care ultrasound training during the COVID-19 pandemic
Source: Ultrasound J. 2021 Sep 6;13:39. doi: 10.1186/s13089-021-00242-6 (PMC8419826; doi:10.1186/s13089-021-00242-6)
Supplement: Supplementary file 1 — Additional file 1: Table S1. Two-day In-person POCUS Course Agenda. Table S2. Four-week Tele-ultrasound POCUS Course Agenda. Table S3. In-person Course Scanning Session Objectives. Table S4. Tele-ultrasound Course Scanning Session Objectives. File S1. Pre- and Post-course Knowledge Test. File S2. CME Course Evaluation. File S3. Tele-ultrasound Course Evaluation. Table S5. Characteristics of Learners and Faculty. Table S6. Tele-ultrasound Course Evaluations by Learners and Faculty. Table S7. Faculty Evaluation of the Tele-ultrasound Course. [file 13089_2021_242_MOESM1_ESM.pdf]

Supplemental Material

Supplemental Table 1. Two-day In-person POCUS Course Agenda

Supplemental Table 2. Four-week Tele-ultrasound POCUS Course Agenda

Supplemental Table 3. In-person Course Scanning Session Objectives

Supplemental Table 4. Tele-ultrasound Course Scanning Session Objectives

Supplemental File 5. Pre- and Post-course Knowledge Test

Supplemental File 6. CME Course Evaluation

Supplemental File 7. Tele-ultrasound Course Evaluation

Supplemental Table 8. Characteristics of Learners and Faculty

Supplemental Table 9. Tele-ultrasound Course Evaluations by Learners and Faculty

Supplemental Table 10. Faculty Evaluation of the Tele-ultrasound Course

Supplemental Table 1. Two-day In-person POCUS Course Agenda

Day 1

Time	Group 1	Group 2
0700 – 0730	Registration & Breakfast	
0730 – 0800	Point-of-care Ultrasound Pre-test	
0800 – 0810	Welcome & Course Overview	
0810 – 0830	Getting Started: Ultrasound Equipment & Knobology	
0830 – 0915	Focused Cardiac Ultrasound Lecture	
0915 – 0930	*Break & Divide into 2 groups*	
0930 – 1025	Table 1: Parasternal long- & short-axis views/ Apical 4-chamber view *(30 min)*<br>Table 2: Subcostal 4-chamber view & IVC/ Apical 4-chamber view *(25 min)*	Practice Interpretation: Cardiac Images
1025 – 1030	*Rotate Groups 1 & 2*	
1030 – 1125	Practice Interpretation: Cardiac Images	Table 1: Parasternal long- & short-axis views/ Apical 4-chamber view *(30 min)*<br>Table 2: Subcostal 4-chamber view & IVC/ Apical 4-chamber view *(25 min)*
1125 – 1130	*Reconvene in Lecture Hall*	
1130 – 1200	DVT and Basic Vascular Ultrasound Lecture	
1200 – 1300	*Lunch*	
1300 – 1325	Lung & Pleural Ultrasound Lecture	
1325 – 1330	*Divide into 2 groups*	
1330 – 1410	Table 1: Lungs & pleura / LE DVT *(20 min)*<br>Table 2: LE DVT / Lungs & pleura *(20 min)*	Practice Interpretation: Lung, Pleural, and Vascular Images
1410 – 1415	*Rotate Groups 1 & 2*	
1415 – 1500	Practice Interpretation: Lung, Pleural, and Vascular Images	Table 1: Lungs & pleura / LE DVT *(20 min)*<br>Table 2: LE DVT / Lungs & pleura *(20 min)*
1500 – 1515	*Break then Reconvene in Lecture Hall*	
1515 – 1555	Procedures: Vascular Access, Paracentesis, Thoracentesis, Lumbar Puncture Lecture	
1555 – 1600	*Divide into 2 groups*	
1600 – 1640	Table 1: Thoracentesis, Paracentesis *(20 min)*<br>Table 2: Lumbar puncture *(20 min)*	Table 1: CVC & PIV *(20 min)*<br>Table 2: Practice Image Interpretation *(20 min)*
1640 – 1645	*Rotate Groups 1 & 2*	
1645 – 1730	Table 1: CVC & PIV *(20 min)*<br>Table 2: Practice Image Interpretation *(20 min)*	Table 1: Thoracentesis, Paracentesis *(20 min)*<br>Table 2: Lumbar puncture *(20 min)*
1730	*Adjourn*	

Day 2

Time	Group 1	Group 2
0700 – 0800	*Breakfast*	
0800 – 0840	Abdominal Ultrasound Lecture	
0840 – 0845	*Divide into 2 groups*	
0845 – 0945	Table 1: RUQ (Gallbladder & Kidney) & LUQ (Spleen & Kidney) *(30 min)*<br>Table 2: Aorta & Pelvis (Bladder & Uterus/Prostate) *(30 min)*	Practice Interpretation: Abdominal Images
0945 – 1000	*Break & Rotate Groups 1 & 2*	
1000 – 1100	Practice Interpretation: Abdominal Images	Table 1: RUQ (Gallbladder & Kidney) & LUQ (Spleen & Kidney) *(30 min)*<br>Table 2: Aorta & Pelvis (Bladder & Uterus/Prostate) *(30 min)*
1100 – 1105	*Reconvene in Lecture Hall*	
1105 – 1135	Cardiac Arrest Lecture	
1135 – 1200	Skin, Soft Tissues, & Joints Lecture	
1200 – 1300	*Lunch*	
1300 – 1315	Imaging Pearls & Pitfalls Lecture	
1315 – 1320	*Divide into 2 groups*	
1320 – 1430	Practice Interpretation: Cases & Abnormal Images	Review Session:<br>Cardiac Ultrasound, Lungs & Abdomen, Vascular / LE DVT, Pediatrics, US-guided Procedures, and Patients
1430 – 1445	*Break & Rotate Groups 1 & 2*	
1445 – 1600	Review Session:<br>Cardiac Ultrasound, Lungs & Abdomen, Vascular / LE DVT, Pediatrics, US-guided Procedures, and Patients	Practice Interpretation: Cases & Abnormal Images
1600 – 1605	*Reconvene in Lecture Hall*	
1605 – 1630	Ultrasound Program Development	
1630 – 1700	Questions & Answers<br>Faculty Panel Discussion	
1700 – 1730	Post-course test & Course Evaluation	
1730	*Adjourn*	

Supplemental Table 2. Four-week Tele-ultrasound POCUS Course Agenda

Pre-course	
**Time**	**Self-directed reading performed 1-2 months prior to the course**
**60 minutes**	Point-of-care Ultrasound Pre-test
**90 minutes**	Chapter 9: Lung Ultrasound
**90 minutes**	Chapter 14: Cardiac Ultrasound Technique
**90 minutes**	Chapter 24: Peritoneal Free Fluid
**90 minutes**	Chapter 34: Lower Extremity DVT
**60 minutes**	Chapter Review Questions

Week 1	
**Time**	**Group Lectures: Tuesday, January 26th**
**1300 – 1310**	Welcome & Course Overview
**1310 – 1330**	Getting Started: Ultrasound Equipment & Knobology
**1330 – 1415**	Focused Cardiac Ultrasound Lecture
**1415 – 1430**	Break
**1445 – 1515**	Lung/Pleural Ultrasound Lecture
**1515 – 1545**	DVT and Basic Vascular Ultrasound Didactic
**1545 – 1630**	Abdominal Ultrasound Lecture
**1630 – 1715**	Practice Interpretation: Cardiac, Lung, DVT, Abdominal Images
	**Individual Hands-on Scanning Session**
**90 minutes**	Hands-on training session: Cardiac, Lung, DVT, Abdominal Views

Week 2	
**Time**	**Group Lectures**
**1400 – 1500**	Practice Interpretation: Cardiac Images
**1500 – 1600**	Procedures: Vascular Access, Paracentesis, Thoracentesis, Lumbar Puncture
**1600 – 1630**	Skin, Soft Tissues, & Joints Lecture
	**Individual Hands-on Scanning Session**
**90 minutes**	Hands-on training session: Cardiac, Lung, DVT, Abdominal Views

Week 3	
**Time**	**Group Lectures**
**1400 – 1500**	Practice Interpretation: POCUS cases
**1500 – 1600**	Cardiac Arrest Lecture
	**Individual Hands-on Scanning Session**
**90 minutes**	Hands-on training session: Central & peripheral venous access, Thoracentesis, Paracentesis, & Lumbar puncture

Week 4	
**Time**	**Group Lectures**
**1400 – 1500**	Practice Interpretation: POCUS Image Review & Cases
**1500 – 1530**	Ultrasound Program Development
**1530 – 1600**	Questions & Answers<br>Faculty Panel Discussion – All Faculty
	**Individual Hands-on Scanning Session**
**90 minutes**	Hands-on training session: Cardiac, Lung, DVT, Abdominal Views
**60 minutes**	Post-course test & Course Evaluation

Supplemental Table 3. In-person Course Scanning Session Objectives

<table>
<tr><th>TABLE 1</th><th>TABLE 2</th></tr>
<tr><td colspan="2">Day 1: CARDIAC (30 min rotation)</td></tr>
<tr><td>• Parasternal long-axis view: Identify RV, LV, septum, MV, AV, aorta, LA, DTA

• Parasternal short-axis view (mid-LV level): Identify RV and LV walls (septal, inferior, lateral, anterior)

• Apical 4-chamber view: Identify 4 chambers, TV, MV. (Optional: demonstrate A5C view by tilting anteriorly)</td><td>• Subcostal 4-chamber view: Identify liver and 4 chambers, TV, MV. (Optional: demonstrate subcostal short-axis view by rotating 90 deg counterclockwise)

• Subcostal IVC: Identify IVC and RA junction

• Apical 4-chamber view: Identify 4 chambers, TV, MV. (Optional: demonstrate A5C view by tilting anteriorly)</td></tr>
<tr><td colspan="2">Day 1: LUNG & DVT (20 min rotation)</td></tr>
<tr><td>• Anterior Chest Wall: Start with linear probe to show pleural sliding and identify chest wall, rib (actual rib), rib shadow. Demonstrate sliding with M-mode.

• Anterior Chest Wall: Switch to phased-array probe to see sliding and A-lines on anterior and lateral chest wall

• Costophrenic Recess: Identify liver, diaphragm, and right costophrenic recess. Point our curtain sign, mirror image and absence of spine sign. (Optional: evaluate right costophrenic recess)</td><td>• Thigh: Start at inguinal crease and slide down thigh to identify: CFV; CFV-GSV; CFV-lateral perforators; CFA split into SFA & DFA; CFV split into FV & DFV

• Popliteal area: Identify PV & PA

(Optional: Scan both lower extremities)</td></tr>
<tr><td colspan="2">Day 1: PROCEDURES (20 min rotation)</td></tr>
<tr><td>• Thoracentesis (live model): Appreciate chest wall anatomy using linear probe as if marking a site for thoracentesis. Identify rib, intercostal artery (more exposed closer to spine and must tilt ~60 deg if using color Doppler with low flow setting), and practice measuring depth. Show exactly how and where you would mark a patient.

• Pleural effusion (simulation model): Demonstrate pleural effusion on simulation model. (Optional: insert needle to perform thoracentesis. Consider practicing real-time guidance to spice it up).

• Paracentesis (live model): Appreciate abdominal wall anatomy. Start in transverse plane and slide laterally. Identify linea alba, rectus abdominus muscle, inferior epigastrics along postero-lateral edge of rectus abdominus (slide toward femoral vessels if difficulty finding inferior epigastrics and use color Doppler on medium flow setting),</td><td>• Lumbar puncture mapping (live model): With model sitting upright, use linear or curvilinear probe in transverse plane starting low over sacrum (as if you had no landmarks). Slide cephalad and identify lumbar spinous processes sequentially until L2. Rotate probe 90 clockwise into a longitudinal plane. Identify interspinous spaces while sliding caudal: L2-L3, L3-L4, and L4-L5. Assess width of interspinous spaces. Demonstrate how to mark midline and interspinous spaces. Slide few millimeters laterally in a longitudinal orientation to identify the ligamentum flavum and measure skin-ligamentum flavum distance.

(Optional: Practice lumbar puncture mapping and needle insertion on simulation models.)</td></tr>
</table>

<table>
<tr><td>muscular aponeurosis, 3 muscle layers (transversus abdominis, internal and external obliques)

• Ascites (simulation model): Demonstrate peritoneal free fluid and loops of bowel on simulation model (Optional: insert needle to perform paracentesis. Consider practicing real-time guidance to spice it up).</td><td></td></tr>
<tr><td colspan="2"></td></tr>
<tr><th colspan="2">Day 2: ABDOMEN (30 min rotations)</th></tr>
<tr><td>• RUQ: Identify diaphragm, liver, and Morison's pouch then slide inferiorly to assess kidney in longitudinal plane and tilt (fan) through the kidney.

• Gallbladder: Identify gallbladder fundus, neck, and portal triad (add color Doppler to differentiate common bile duct, hepatic artery, portal vein)

• LUQ: Identify diaphragm, spleen, and kidney. Point out left subdiaphragmatic space where free fluid collects. Slide inferiorly to assess kidney in longitudinal plane and tilt (fan) through the kidney.</td><td>• Aorta: Start in epigastric area in transverse plane and slide inferiorly to identify as many branches as possible (celiac trunck, SMA, renal arteries, renal veins and IVC) until bifurcation into common iliacs. Acquire 1 long-axis view of upper or mid-aorta.

• Pelvis: Identify bladder, prostate or uterus, rectum in both transverse and longitudinal planes. Practice measuring bladder dimensions and calculating volume. (Optional: Use color or power Doppler to look for ureteral jets.)</td></tr>
<tr><th colspan="2">Day 2: REVIEW (30 min rotations)</th></tr>
<tr><td colspan="2">• *Faculty stay at one station and learners wander to different stations to practice.

• <u>Stations</u>: Cardiac, lung/abdomen, and vascular/LE DVT. Notify Elizabeth which models have good views for each station. Goal is to practice acquiring all views taught during the course.

• <u>Simulation models</u>: ALL are available for practice, including vascular access models in Iberian C

• <u>Patients</u>: We anticipate 4 patients arriving (3 cirrhotics; 1 heart failure)

• <u>Pediatrics</u>: We will have 2 children ages 8-12 for the pediatrics stations</td></tr>
</table>

Supplemental Table 4. Tele-ultrasound Course Scanning Session Objectives

**SESSION 1: INTRODUCTION**	
OBJECTIVES	• Become familiar with basic operation of the ultrasound machine (probe selection, exam type, depth, gain, saving video clips)<br>• Practice acquiring standard views and identifying key structures in this order of priority (see "Skills List" below):<br>o Lower extremity deep venous thrombosis exam<br>o Lung exam<br>o Cardiac exam<br>o Abdominal exam
**SESSION 2: CARDIAC**	
OBJECTIVES	1. Practice acquiring the 5 standard focused cardiac ultrasound views: parasternal long- and short-axis views, apical 4-chamber view, and subcostal 4-chamber and IVC views (see "Skills List" below).<br>2. Provide specific guidance and pearls for image optimization of the cardiac views, such as left lateral decubitus position for parasternal views, tilting probe to obtain different PSAX views, gentle breath hold for S4C view, rocking probe to center septum in A4C view.<br>3. Practice acquiring standard views and identifying key structures in this order of priority (see "Skills List" below):<br>o Abdominal exam<br>o Lung exam<br>o Lower extremity deep venous thrombosis exam
**SESSION 3 : ABDOMEN**	
OBJECTIVES	1. Practice acquiring the standard abdominal ultrasound views on the live model: RUQ, gallbladder, LUQ, aorta, and pelvis (see "Skills List" below). If the gallbladder was difficult to visualize, make note in the "feedback" column of the scheduling spreadsheet and the next faculty can attempt to demonstrate this skill.<br>2. Practice acquiring standard views and identifying key structures in this order of priority (see "Skills List" below):<br>o Cardiac exam<br>o Lower extremity deep venous thrombosis exam<br>o Lung exam

**SESSION 4: PROCEDURES & REVIEW**	
OBJECTIVES	1. Using the live model, practice ultrasound site marking for the following procedures:<br><br>• Thoracentesis: Appreciate chest wall anatomy using linear probe as if marking a site for thoracentesis. Identify rib, intercostal artery (more exposed closer to spine and must tilt ~60 deg if using color Doppler with low flow setting), and practice measuring depth. Show exactly how and where you would mark a patient.<br><br>• Paracentesis: Appreciate abdominal wall anatomy. Start in transverse plane and slide laterally. Identify linea alba, rectus abdominus muscle, inferior epigastrics along postero-lateral edge of rectus abdominus (slide toward femoral vessels if difficulty finding inferior epigastrics and use color Doppler on medium flow setting), muscular aponeurosis, 3 muscle layers (transversus abdominis, internal and external obliques)<br><br>• Lumbar puncture mapping (live model): With model sitting upright, use linear or curvilinear probe in transverse plane starting low over sacrum (as if you had no landmarks). Slide cephalad and identify lumbar spinous processes sequentially until L2. Rotate probe 90 clockwise into a longitudinal plane. Identify interspinous spaces while sliding caudal: L2-L3, L3-L4, and L4-L5. Assess width of interspinous spaces. Demonstrate how to mark midline and interspinous spaces. Slide few millimeters laterally in a longitudinal orientation to identify the ligamentum flavum and measure skin-ligamentum flavum distance.<br><br>2. Using the peripheral IV simulation model, practice real-time needle tip tracking in both transverse and longitudinal planes. All learners will have a Blue Phantom PIV model, needles, and syringes. Encourage learners to continue practicing after the session has ended.<br><br>• Provide clarification and address any questions about ultrasound machine operation and standard POCUS views.<br><br>• Practice acquiring standard views and identifying key structures in this order of priority (see “Skills List” below):<br><br>o Cardiac exam<br>o Abdominal exam<br>o Lower extremity deep venous thrombosis exam<br>o Lung exam
**SKILLS LIST**	
**Lower Extremity DVT Exam**	• Thigh: Start in inguinal crease and slide down the thigh to identify: CFV; CFV-GSV; CFV-lateral perforators; CFA split into SFA & DFA; CFV split into FV & DFV<br><br>• Popliteal area: Identify PV & PA<br><br>(Optional: Scan both lower extremities)

**Abdominal Exam**	• RUQ: Identify diaphragm, liver, and Morison’s pouch then slide inferiorly to assess kidney in longitudinal plane and tilt (fan) through the kidney.<br><br>• Gallbladder: Identify gallbladder fundus, neck, and portal triad (add color Doppler to differentiate common bile duct, hepatic artery, portal vein)<br><br>• LUQ: Identify diaphragm, spleen, and kidney. Point out left subdiaphragmatic space where free fluid collects. Slide inferiorly to assess kidney in longitudinal plane and tilt (fan) through the kidney.<br><br>• Aorta: Start in epigastric area in transverse plane and slide inferiorly to identify as many branches as possible (celiac trunk, SMA, renal arteries, renal veins and IVC) until bifurcation into common iliac veins. Acquire 1 long-axis view of upper or mid-aorta.<br><br>• Pelvis: Identify bladder, prostate or uterus, rectum in both transverse and longitudinal planes. Practice measuring bladder dimensions and calculating volume. (Optional: Use color or power Doppler to look for ureteral jets.)
**Cardiac Exam**	• Parasternal long-axis view: Identify RV, LV, septum, MV, AV, aorta, LA, DTA<br><br>• Parasternal short-axis view (mid-LV level): Identify RV and LV walls (septal, inferior, lateral, anterior)<br><br>• Apical 4-chamber view: Identify 4 chambers, TV, MV. (Optional: demonstrate A5C view by tilting anteriorly)<br><br>• Subcostal 4-chamber view: Identify liver and 4 chambers, TV, MV. (Optional: demonstrate subcostal short-axis view by rotating 90 deg counterclockwise)<br><br>• Subcostal IVC: Identify liver, IVC, hepatic veins, and RA junction
**Lung Exam**	• Anterior Chest Wall: Start with linear probe to show pleural sliding and identify chest wall, rib, rib shadow. Demonstrate sliding with M-mode.<br><br>• Anterior Chest Wall: Switch to phased-array probe to see sliding and A-lines on anterior and lateral chest wall. Practice “bringing out” the A-lines by tilting the probe.<br><br>• Right Costophrenic Recess: Identify liver, diaphragm, spine, and right costophrenic recess. Point out the curtain sign, mirror image and absence of spine sign.<br><br>• Left Costophrenic Recess: Identify spleen, diaphragm, spine, and left costophrenic recess. Appreciate more posterior probe position to visualize left diaphragm. Identify the curtain sign, mirror image and absence of spine sign.

Supplemental File 5. Pre- and Post-course Knowledge Test

Today's Date ______________________

First Name: ______________________

Last Name: ______________________

POCUS Knowledge Test:
- ◯ Pre-course Test
- ◯ Post-course Test

POCUS KNOWLEDGE TEST QUESTIONS

1) When scanning in 2-dimensional ultrasound mode (B-mode), the highest quality ultrasound images are obtained when the ultrasound beam (probe) is _________.

- ◯ Perpendicular to the target structure
- ◯ Parallel to the target structure
- ◯ 60 degrees to the target structure
- ◯ 45 degrees to the target structure

2) To optimize this parasternal long-axis view of the heart, you should:
(Please refer to the image below.)

- ◯ Increase the near field gain only
- ◯ Increase the far field gain only
- ◯ Increase both near and far field gain
- ◯ Decrease the near field gain only
- ◯ Decrease the far field gain only
- ◯ Decrease both near and far field gain

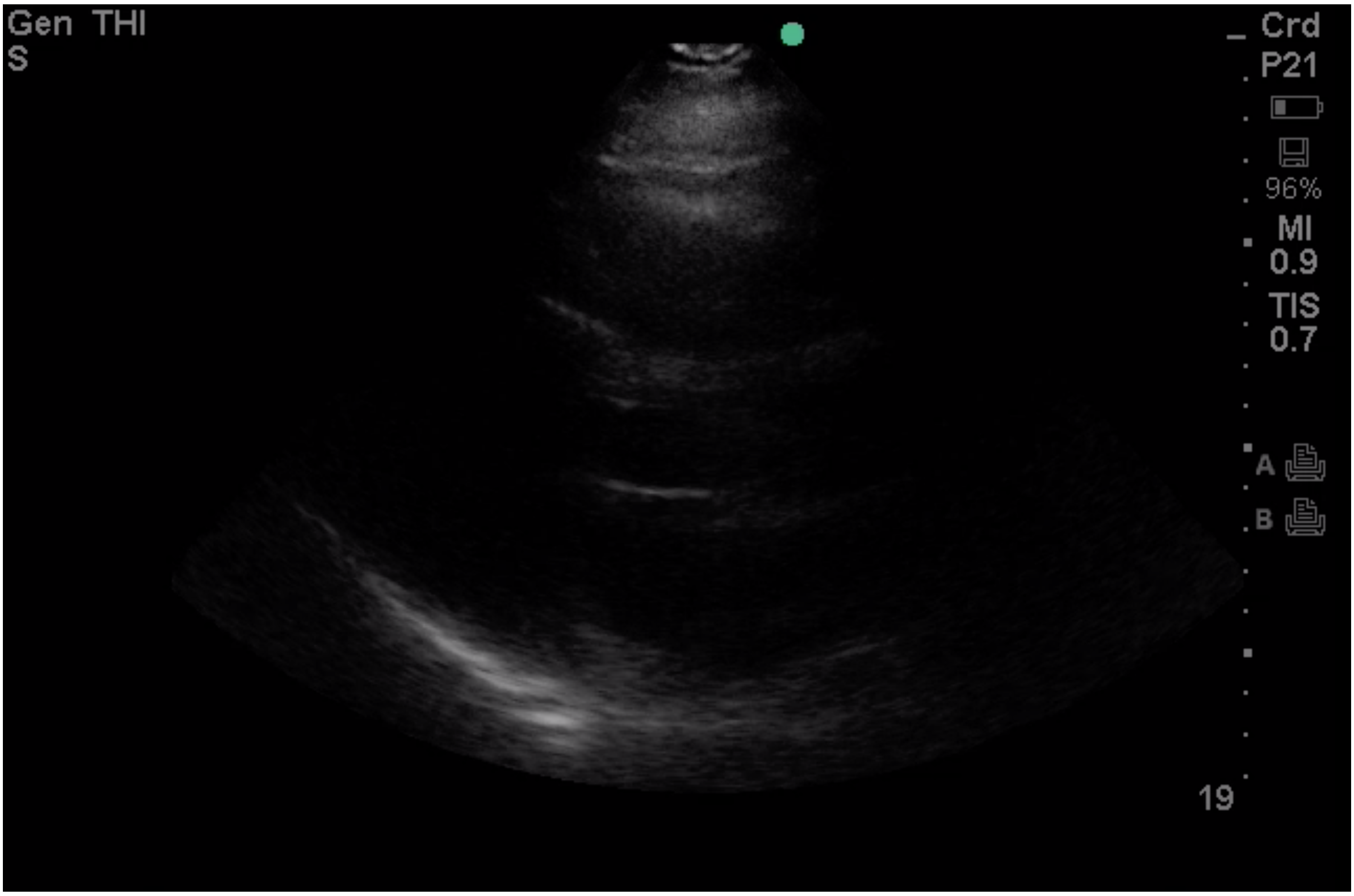

3) To improve the quality of the ultrasound image below, you should:
(Please refer to the image below.)

◯ Increase the near field gain only
◯ Increase the far field gain only
◯ Increase both near and far field gain
◯ Decrease the near field gain only
◯ Decrease the far field gain only
◯ Decrease both near and far field gain

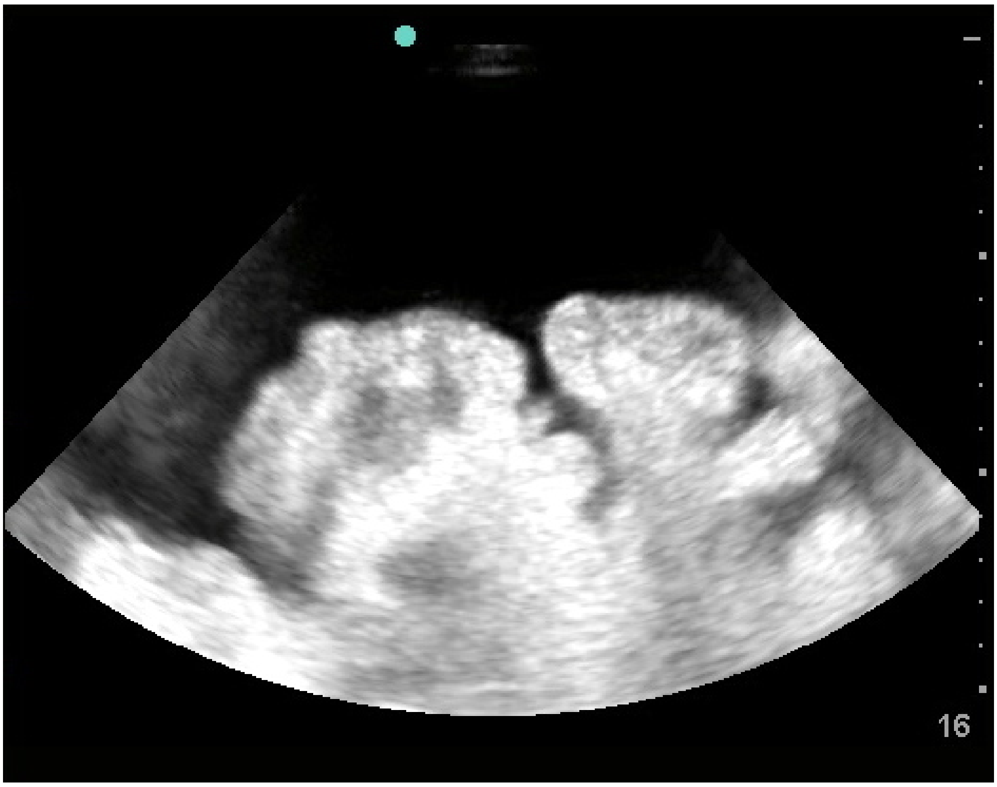

4) Which of these images is most consistent with acute cholecystitis?
(Please refer to the images below.)

◯ A
◯ B
◯ C
◯ D

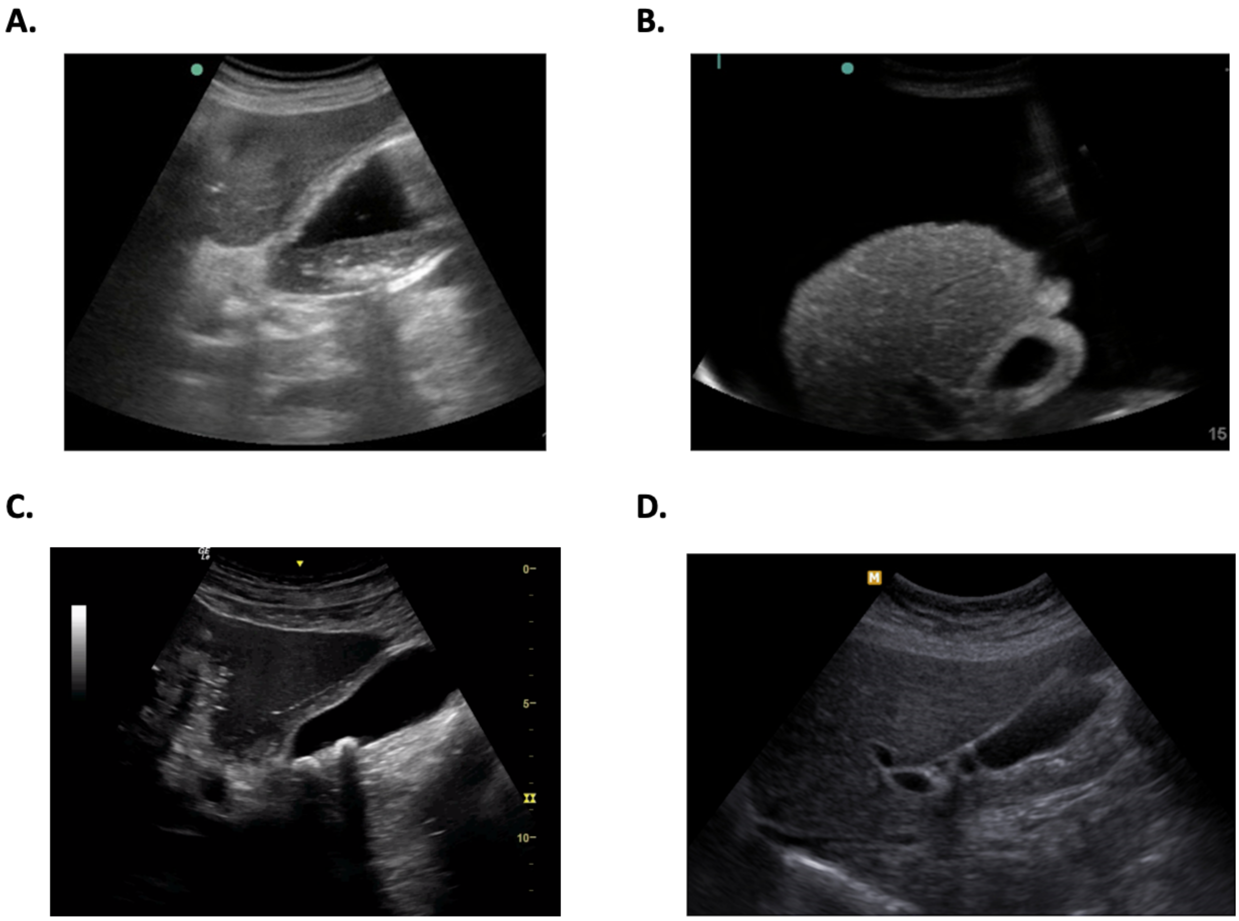

5) Which of these structures is the abdominal aorta?
(Please refer to the image below.)

- ◯ A
- ◯ B
- ◯ C
- ◯ D

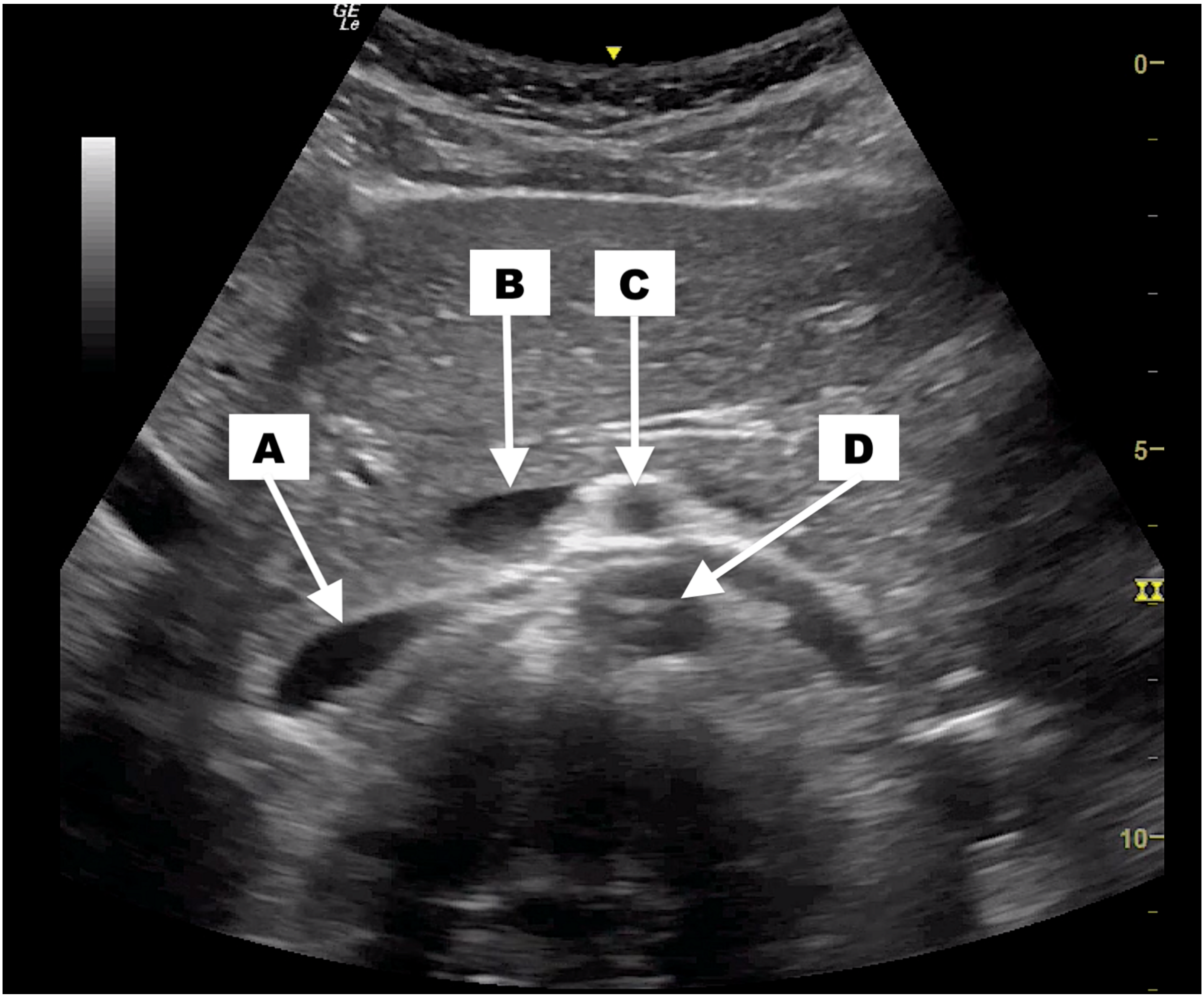

6) This M-mode image of the lung shows ____________________.
(Please refer to the image below.)

◯ Normal lung pattern
◯ Absent lung sliding
◯ Pneumonia
◯ Pleural effusion

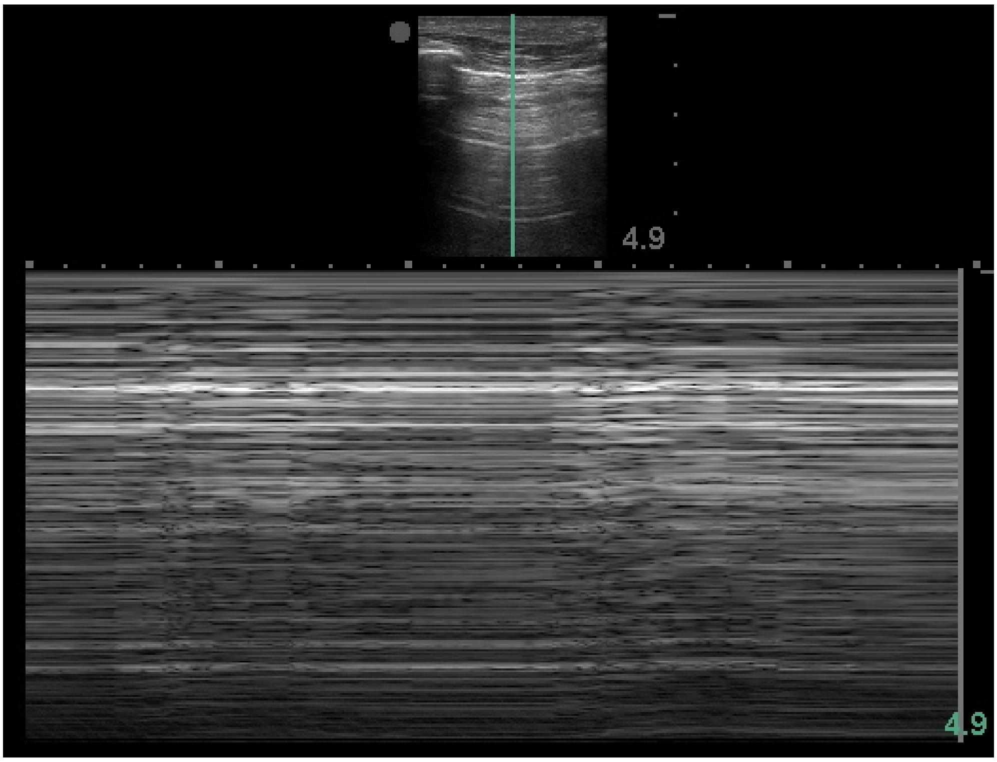

7) What lung ultrasound finding is shown in this image?
(Please refer to the image below.)

◯ A-lines
◯ B-lines
◯ M-lines
◯ Z-lines

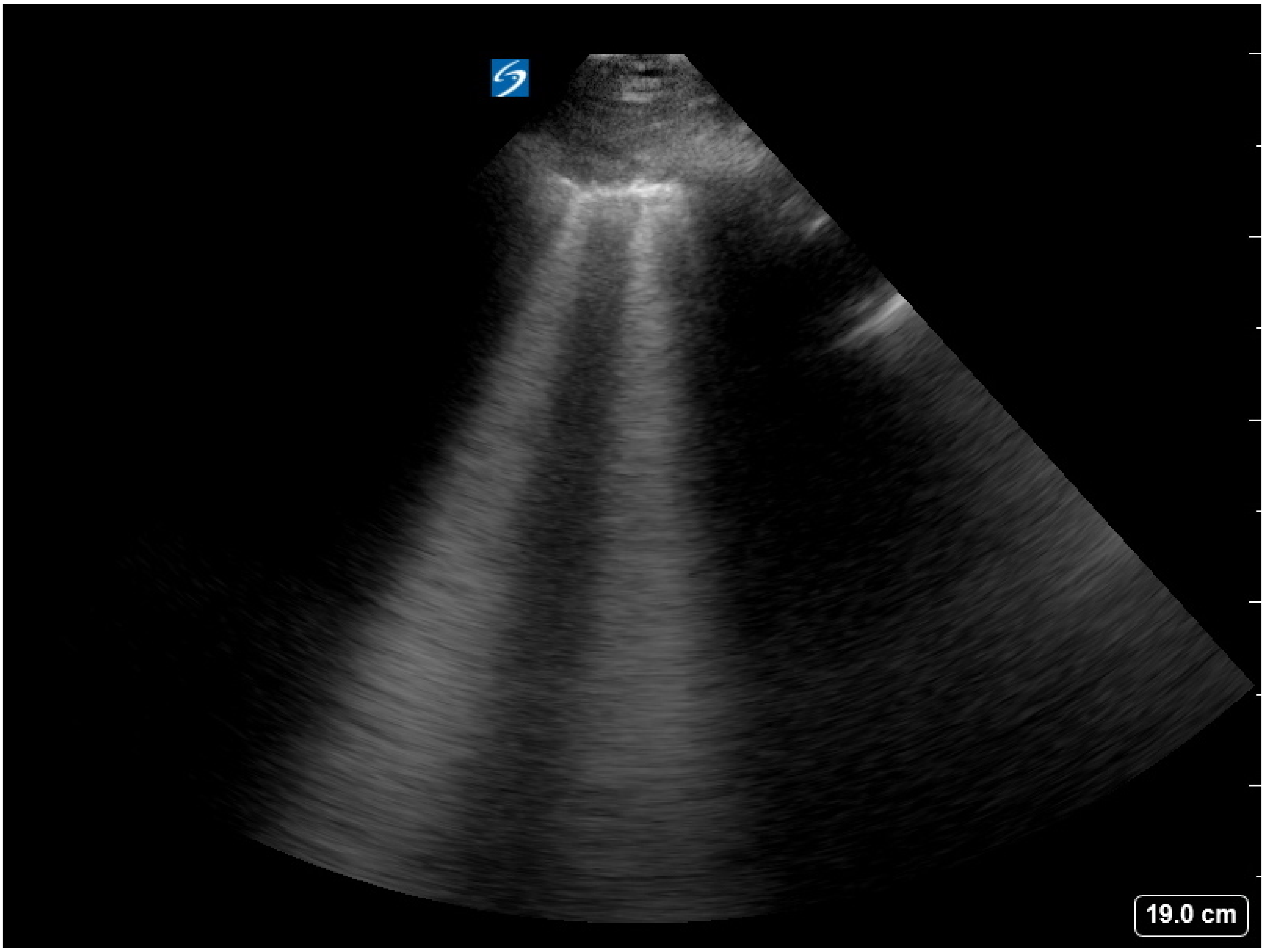

8) Where is free fluid located in this patient based on this image?
(Please refer to the image below.)

◯ Pleural space
◯ Pericardial space
◯ Peritoneal space
◯ Pleural and pericardial space
◯ Pleural and peritoneal space
◯ Pericardial and peritoneal space
◯ Pleural, peritoneal, and pericardial

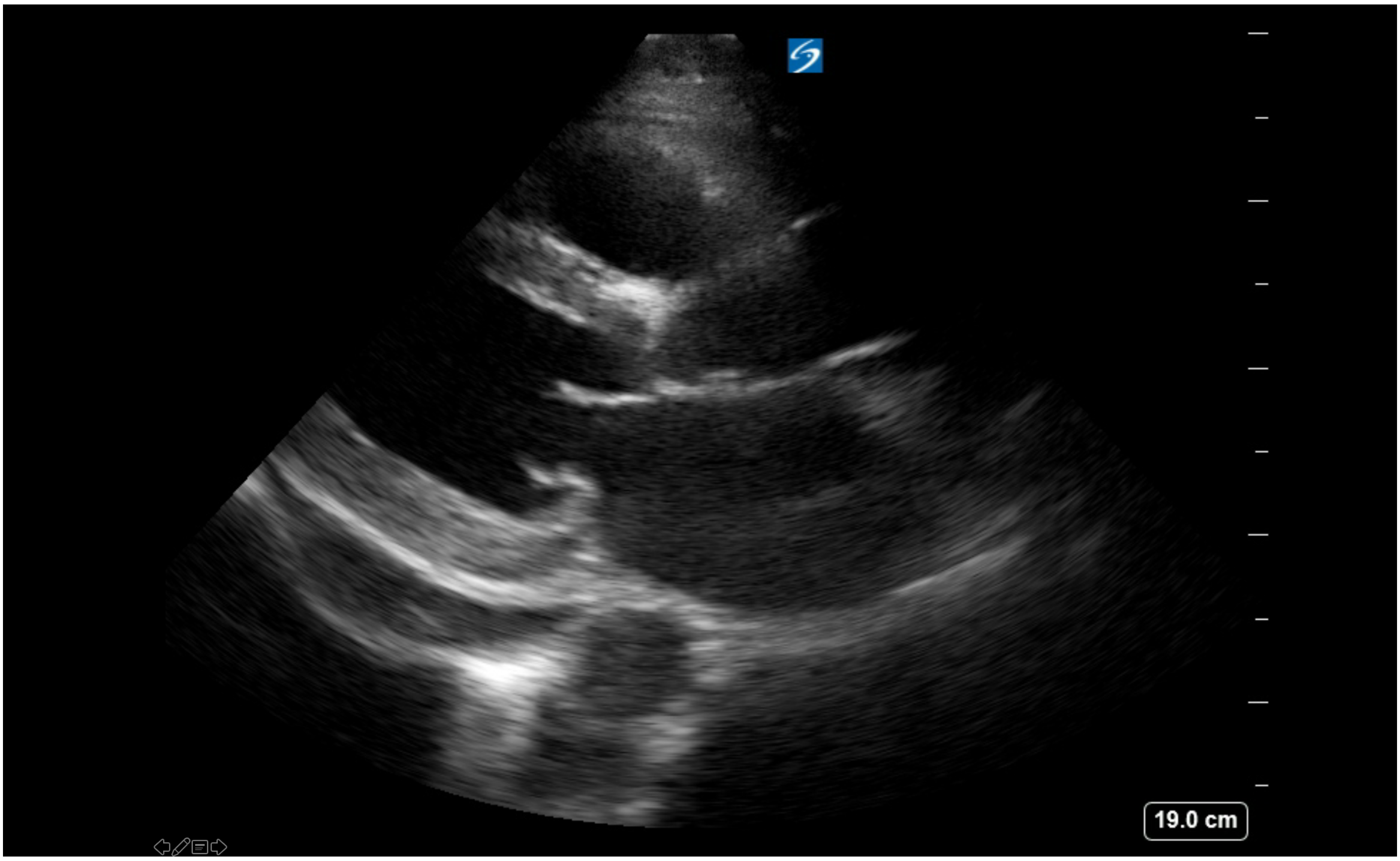

For questions 9-12, match the numbers of the cardiac ultrasound image with the structures listed below.

9) Structure A

◯ Right Ventricle
◯ Right Atrium
◯ Left Ventricle
◯ Left Atrium
◯ Aortic Valve
◯ Mitral Valve
◯ Pericardium
◯ Descending thoracic aorta
◯ Ascending aorta
(Please refer to the image below.)

10) Structure B

◯ Right Ventricle
◯ Right Atrium
◯ Left Ventricle
◯ Left Atrium
◯ Aortic Valve
◯ Mitral Valve
◯ Pericardium
◯ Descending thoracic aorta
◯ Ascending aorta
(Please refer to the image below.)

11) Structure C	◯ Right Ventricle<br>◯ Right Atrium<br>◯ Left Ventricle<br>◯ Left Atrium<br>◯ Aortic Valve<br>◯ Mitral Valve<br>◯ Pericardium<br>◯ Descending thoracic aorta<br>◯ Ascending aorta<br>(Please refer to the image below.)
12) Structure D	◯ Right Ventricle<br>◯ Right Atrium<br>◯ Left Ventricle<br>◯ Left Atrium<br>◯ Aortic Valve<br>◯ Mitral Valve<br>◯ Pericardium<br>◯ Descending thoracic aorta<br>◯ Ascending aorta<br>(Please refer to the image below.)

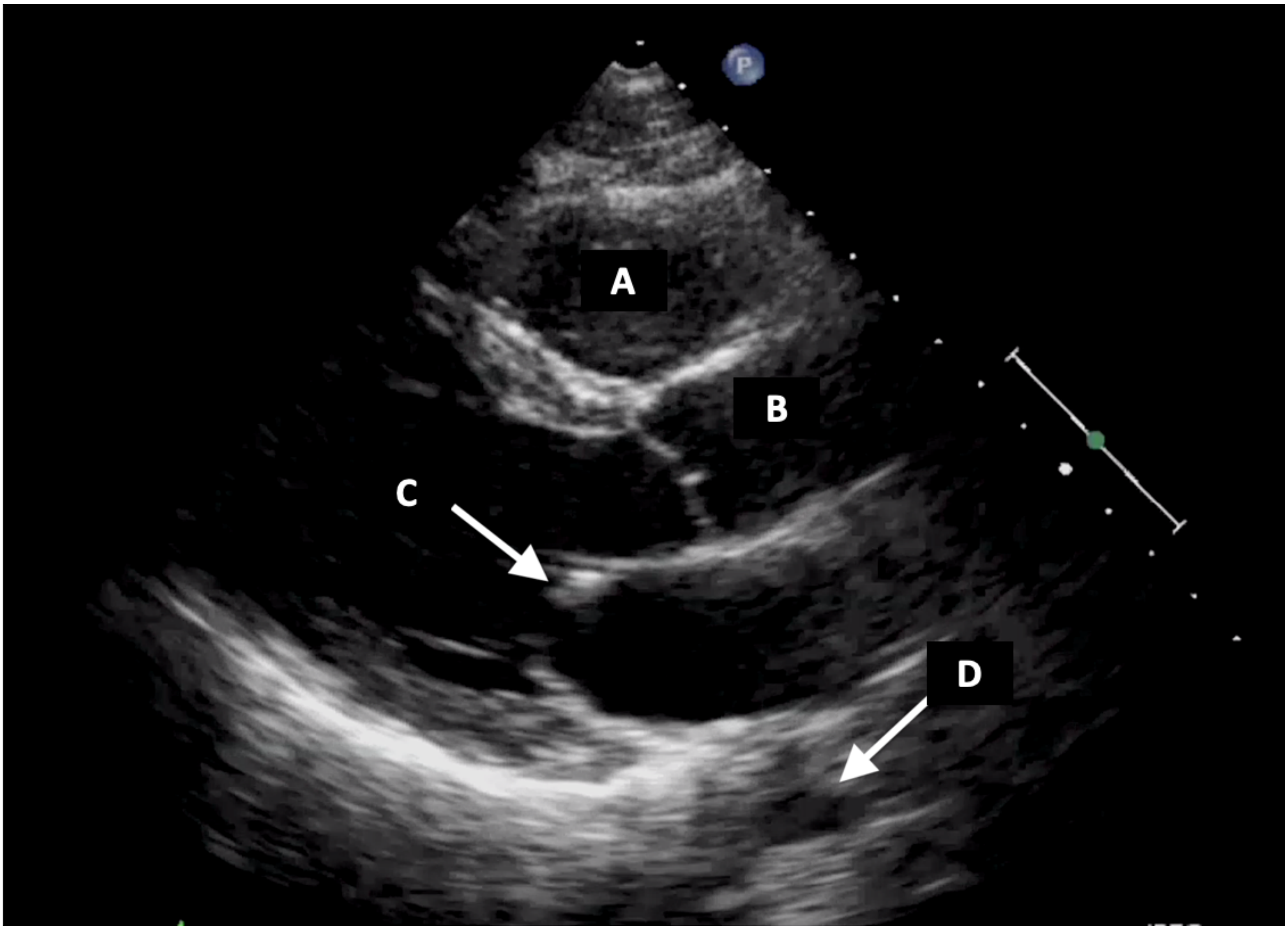

13) In which space(s) does this patient have free fluid based on this subcostal 4-chamber view?
(Please refer to the image below.)

◯ Pleural space
◯ Pericardial space
◯ Peritoneal space
◯ Pleural and pericardial space
◯ Pleural and peritoneal space
◯ Pericardial and peritoneal space
◯ Pleural, peritoneal, and pericardial

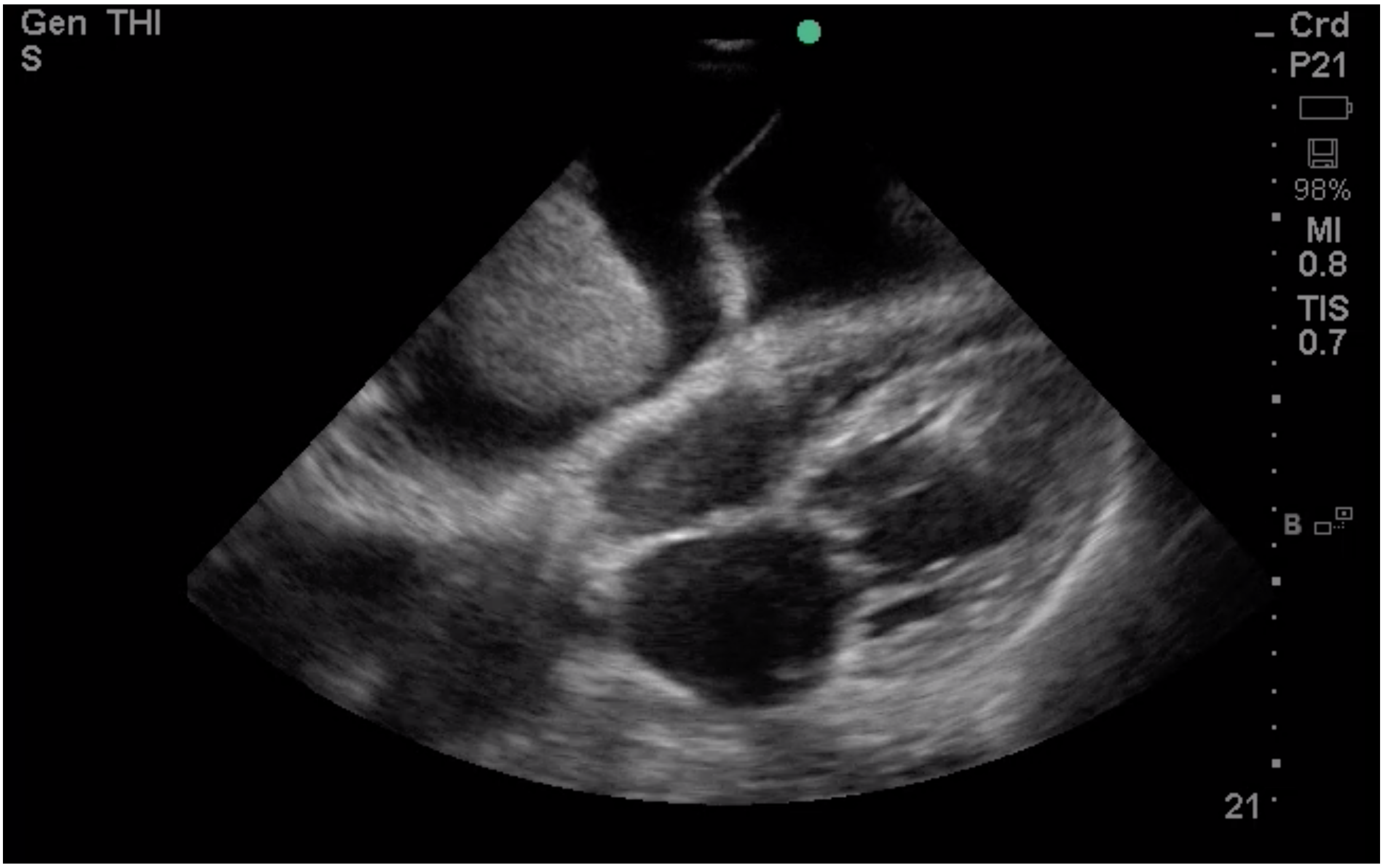

14) What is the most likely cause of shock based on the constellation of findings shown in the videos below?
(Please refer to the videos below.)

◯ Cardiac Tamponade
◯ Massive pulmonary embolism
◯ Hypovolemic shock
◯ Left ventricular dysfunction

15) What is the most likely cause of shock based on the constellation of findings shown in the videos below?
(Please refer to the videos below.)

◯ Cardiac Tamponade
◯ Massive pulmonary embolism
◯ Hypovolemic shock
◯ Left ventricular dysfunction

16) When using Doppler mode, blood flow in a vessel is LEAST likely to be detected if the ultrasound probe is held _______ to the direction of flow.

◯ Perpendicular (90 degrees)
◯ 30 degrees
◯ 45 degrees
◯ 60 degrees
◯ Parallel (0 degrees)

17) In the image shown below, structure A is relatively _______ compared to structure B, and structure C is ________ compared to both A and B.
(Please refer to the image below.)

◯ Anechoic, Hyperechoic
◯ Anechoic, Hypoechoic
◯ Hyperechoic, Anechoic
◯ Hyperechoic, Hypoechoic
◯ Hypoechoic, Anechoic
◯ Hypoechoic, Hyperehoic

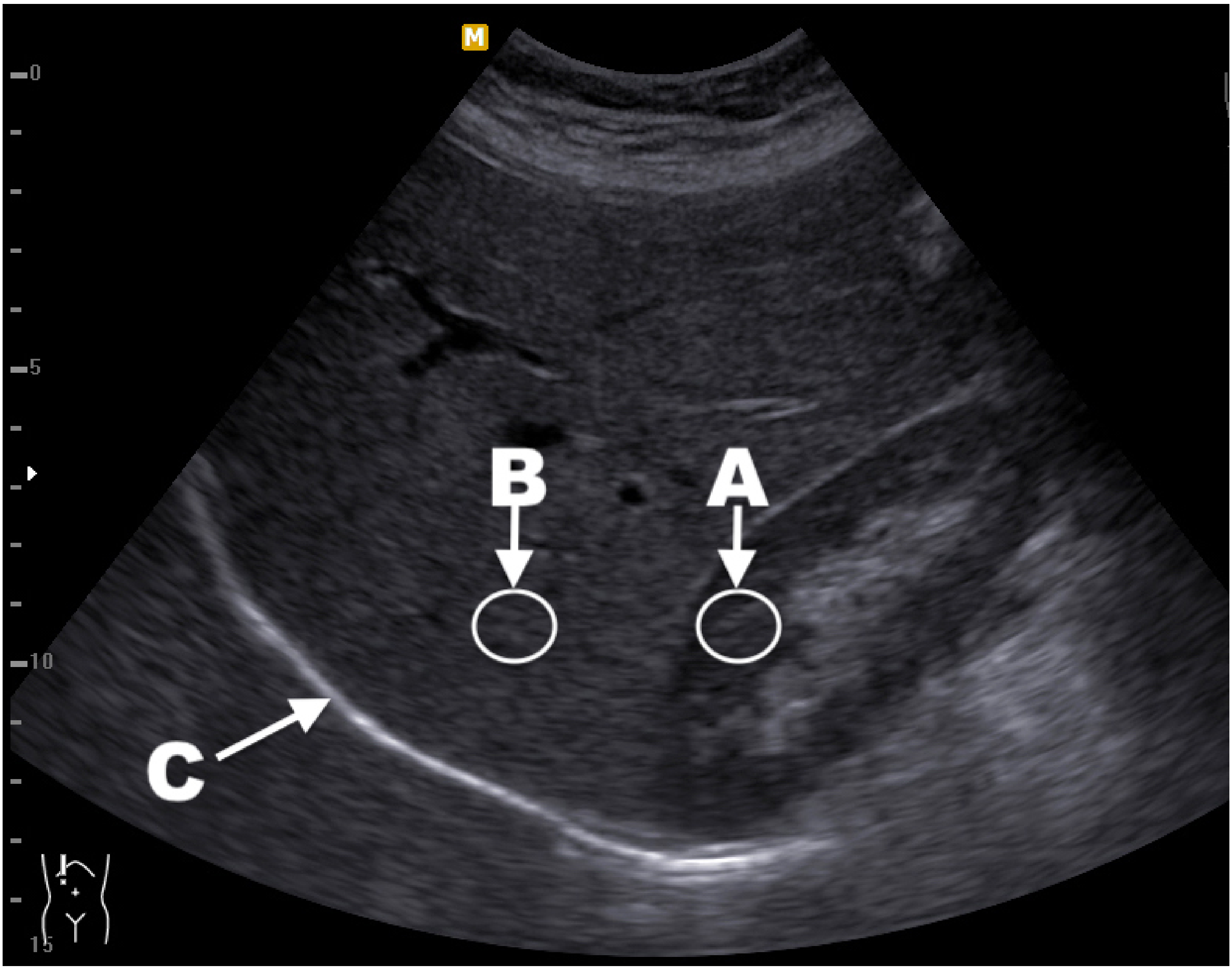

18) What artifact is seen by the red arrows noted in the image below?
(Please refer to the image below.)

◯ Mirror Image Artifact
◯ Posterior Acoustic Enhancement
◯ Reverberation
◯ Edge Artifact
◯ Acoustic Shadowing

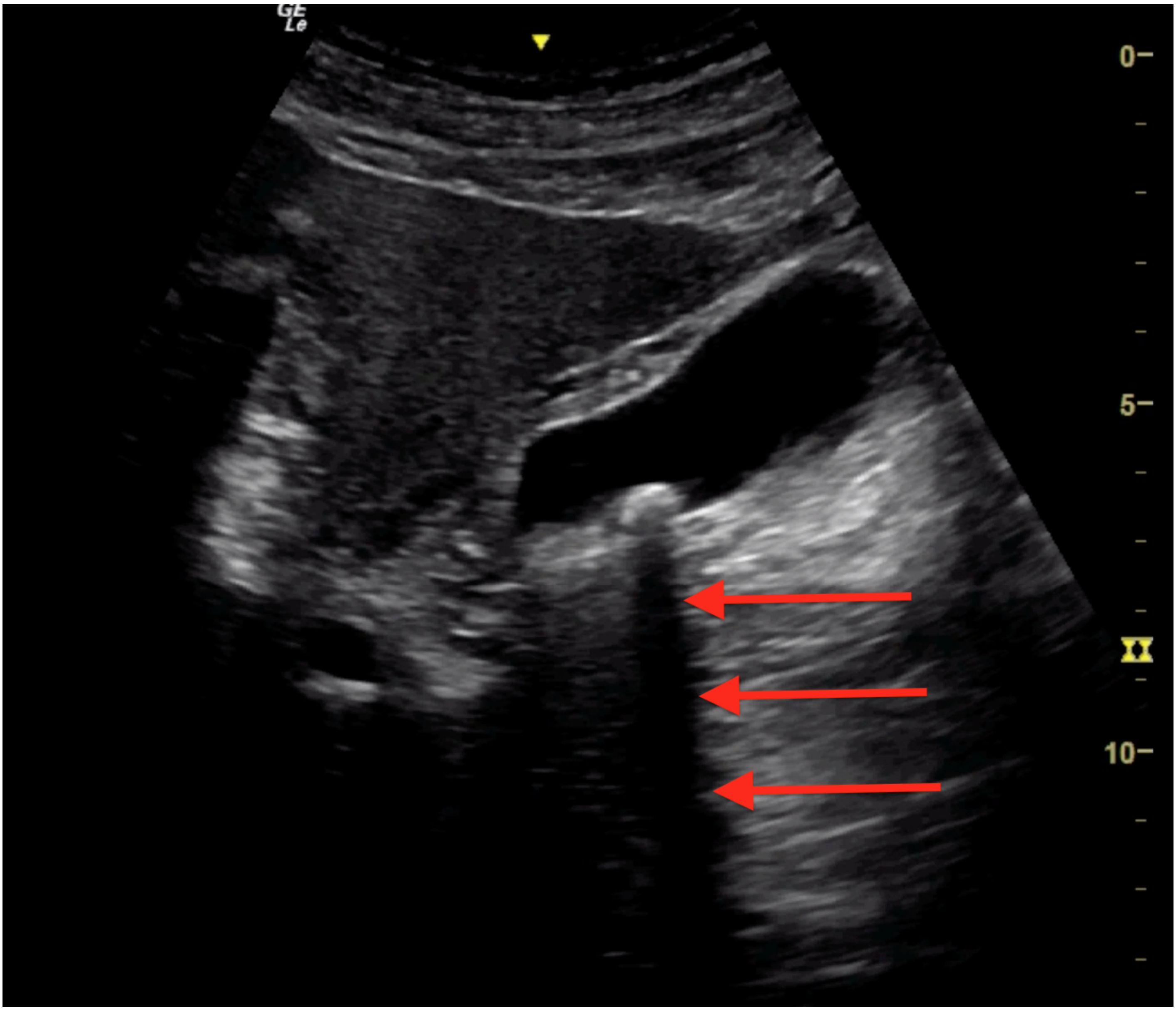

19) In this longitudinal view of the aorta, structure A is the _______ and structure B is ________.
(Please refer to the image below.)

◯ Superior mesenteric artery, celiac trunk
◯ Superior mesenteric artery, inferior mesenteric artery
◯ Celiac trunk, superior mesenteric artery
◯ Celiac trunk, inferior mesenteric artery
◯ Inferior mesenteric artery, celiac trunk
◯ Inferior mesenteric artery, superior mesenteric artery

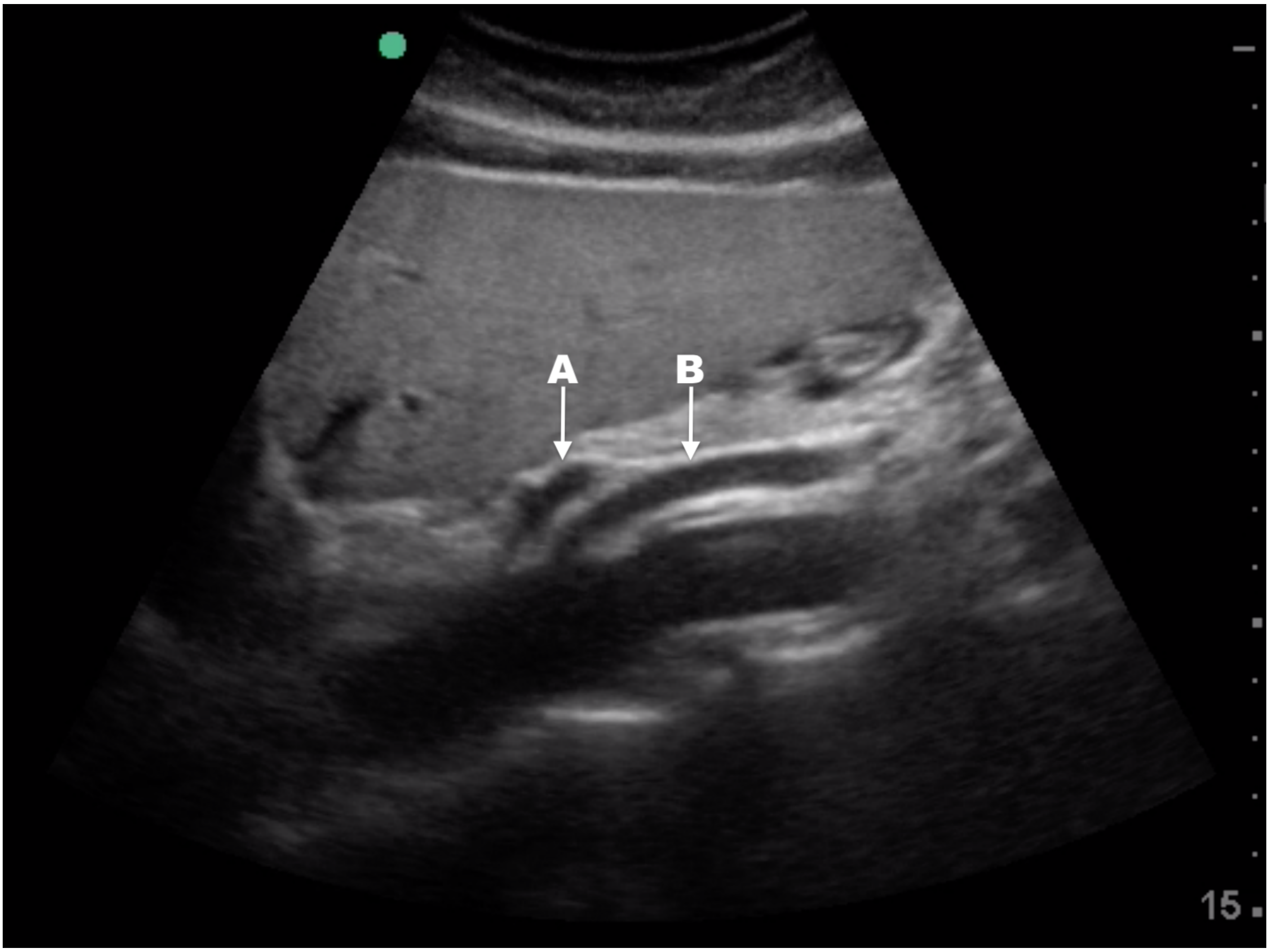

20) Match the structures listed below with the left lower extremity vessels in the image below.

	A	B	C	D
Superficial Femoral Artery	◯	◯	◯	◯
Deep Femoral Artery	◯	◯	◯	◯
Common Femoral Vein	◯	◯	◯	◯
Lateral Perforator Vein	◯	◯	◯	◯

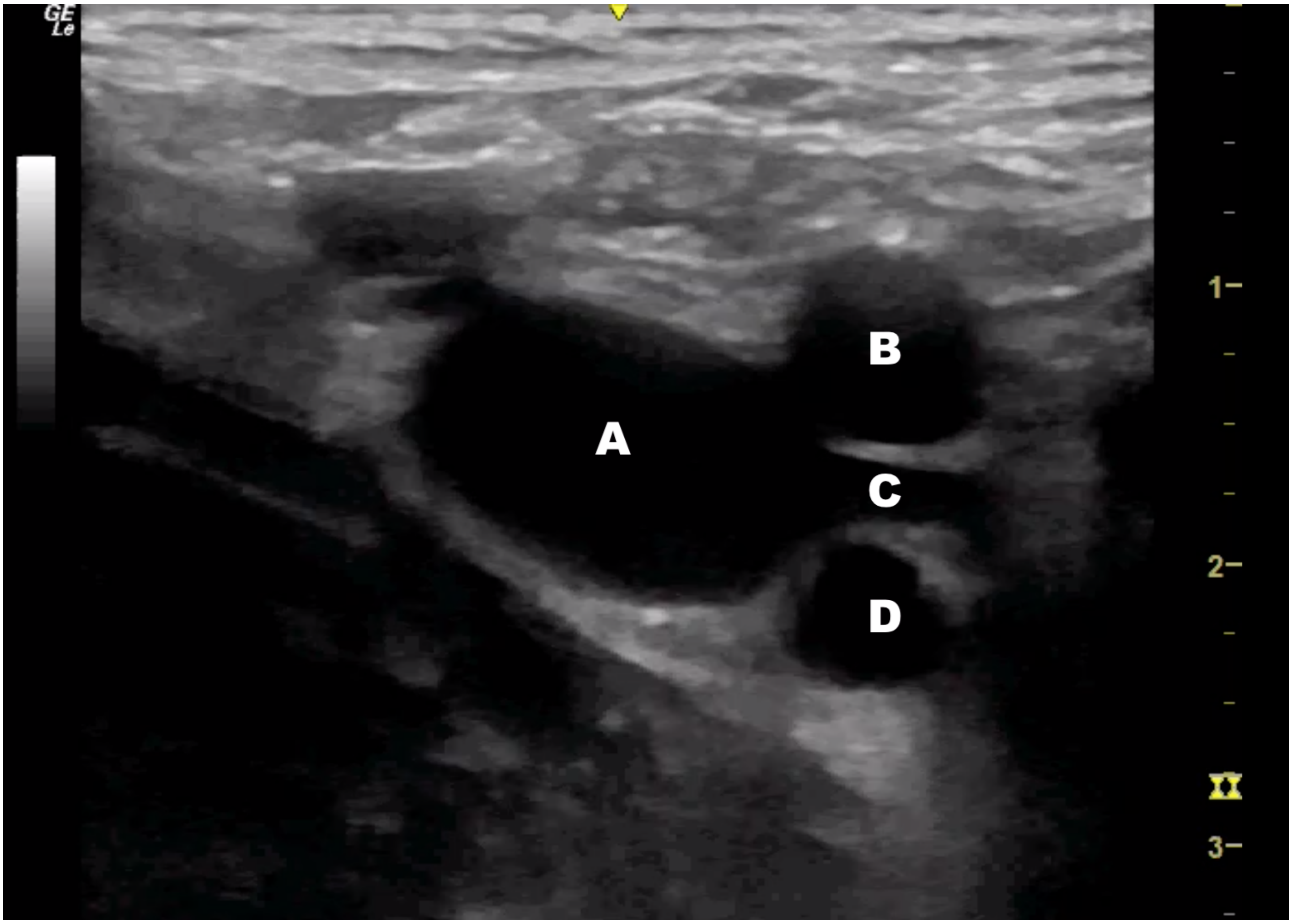

21) What finding is shown in the image below of the kidney?
(Please refer to the image below.)

◯ Mild Hydronephrosis
◯ Moderate Hydronephrosis
◯ Severe Hydronephrosis
◯ Simple renal cyst
◯ Complex renal cyst

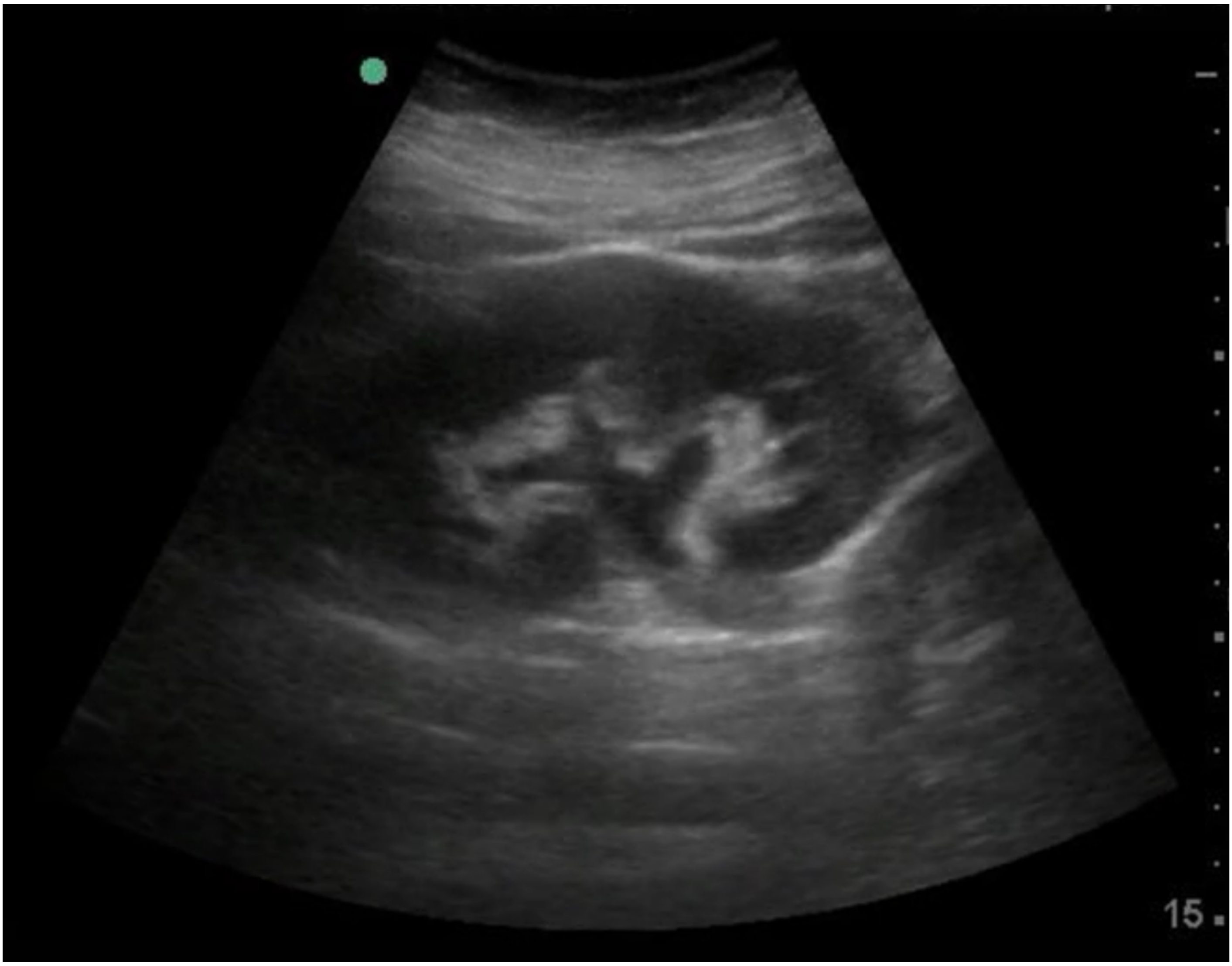

22) What is the approximate bladder volume in the image below?
(Please refer to the image below.)

◯ < 50 mL
◯ 150 mL
◯ 450 mL
◯ 750 mL
◯ >1000 mL

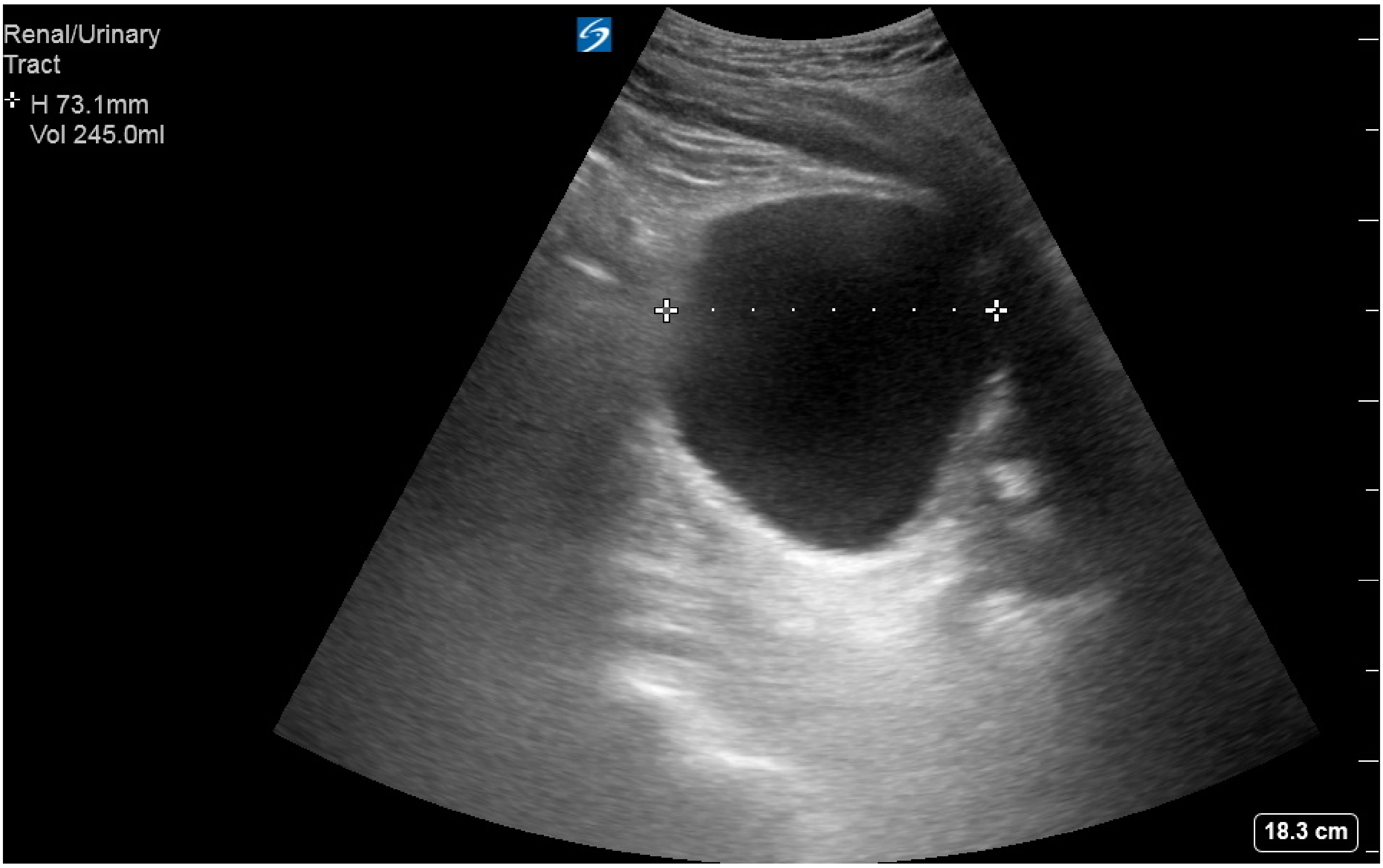
Renal/Urinary
Tract
H 73.1mm
Vol 245.0ml
18.3 cm

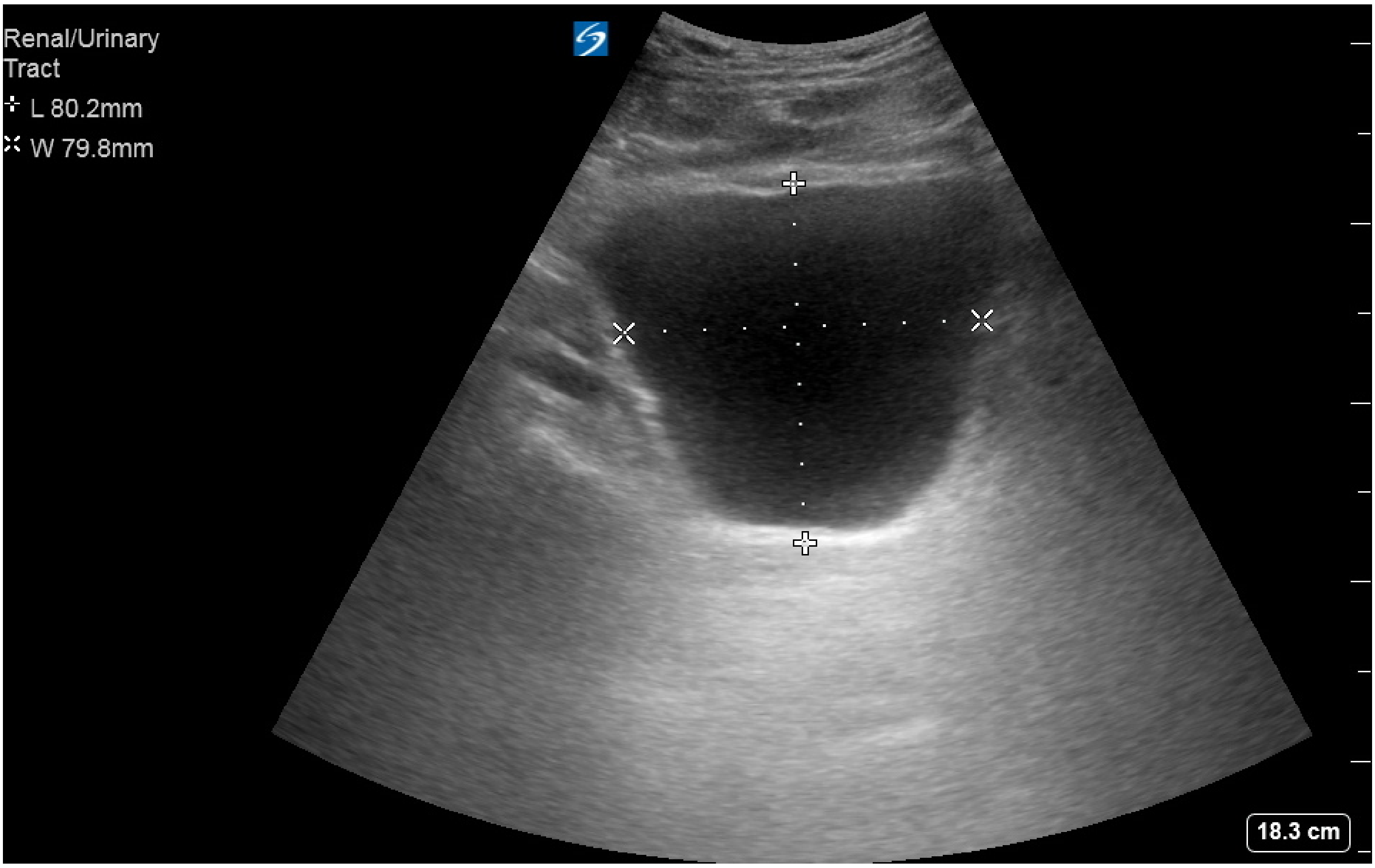
Renal/Urinary
Tract
L 80.2mm
W 79.8mm
18.3 cm

23) When performing an abdominal ultrasound examination to assess for peritoneal free fluid, which space is NOT typically examined with the FAST protocol?

◯ Between the liver and the right kidney
◯ Between the spleen and the diaphragm
◯ Between the uterus and rectum
◯ Between the prostate and bladder

24) Match the letters of this subcostal 4-chamber view with the structures listed below.

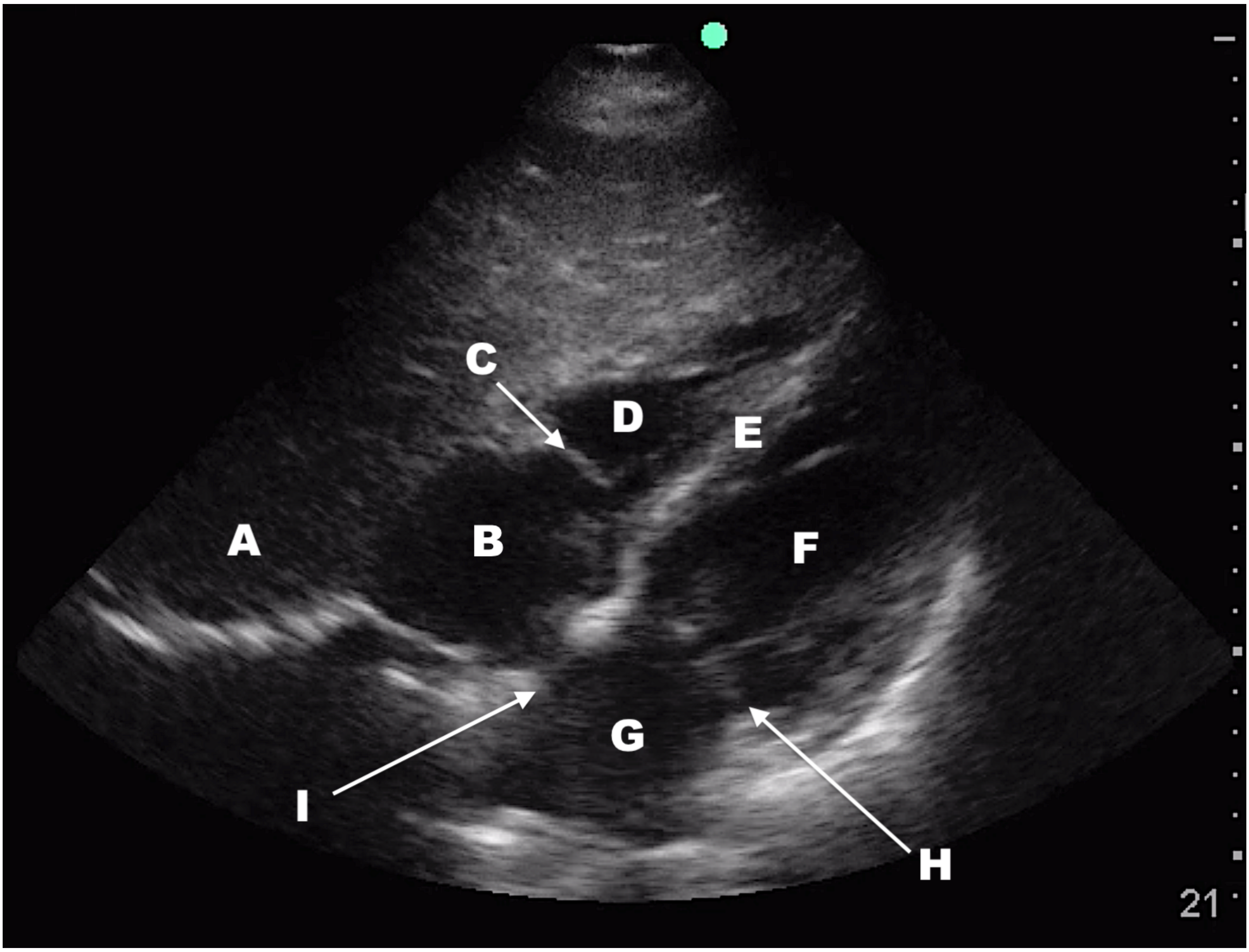

Structure A

◯ Right Ventricle
◯ Liver
◯ Left Atrium
◯ Right Atrium
◯ Left Ventricle
◯ Interventricular Septum
◯ Tricuspid Valve
◯ Mitral Valve
◯ Interatrial Septum

Structure B	◯ Right Ventricle<br>◯ Liver<br>◯ Left Atrium<br>◯ Right Atrium<br>◯ Left Ventricle<br>◯ Interventricular Septum<br>◯ Tricuspid Valve<br>◯ Mitral Valve<br>◯ Interatrial Septum
Structure C	◯ Right Ventricle<br>◯ Liver<br>◯ Left Atrium<br>◯ Right Atrium<br>◯ Left Ventricle<br>◯ Interventricular Septum<br>◯ Tricuspid Valve<br>◯ Mitral Valve<br>◯ Interatrial Septum
Structure D	◯ Right Ventricle<br>◯ Liver<br>◯ Left Atrium<br>◯ Right Atrium<br>◯ Left Ventricle<br>◯ Interventricular Septum<br>◯ Tricuspid Valve<br>◯ Mitral Valve<br>◯ Interatrial Septum
Structure E	◯ Right Ventricle<br>◯ Liver<br>◯ Left Atrium<br>◯ Right Atrium<br>◯ Left Ventricle<br>◯ Interventricular Septum<br>◯ Tricuspid Valve<br>◯ Mitral Valve<br>◯ Interatrial Septum
Structure F	◯ Right Ventricle<br>◯ Liver<br>◯ Left Atrium<br>◯ Right Atrium<br>◯ Left Ventricle<br>◯ Interventricular Septum<br>◯ Tricuspid Valve<br>◯ Mitral Valve<br>◯ Interatrial Septum
Structure G	◯ Right Ventricle<br>◯ Liver<br>◯ Left Atrium<br>◯ Right Atrium<br>◯ Left Ventricle<br>◯ Interventricular Septum<br>◯ Tricuspid Valve<br>◯ Mitral Valve<br>◯ Interatrial Septum

Structure H	◯ Right Ventricle<br>◯ Liver<br>◯ Left Atrium<br>◯ Right Atrium<br>◯ Left Ventricle<br>◯ Interventricular Septum<br>◯ Tricuspid Valve<br>◯ Mitral Valve<br>◯ Interatrial Septum
Structure I	◯ Right Ventricle<br>◯ Liver<br>◯ Left Atrium<br>◯ Right Atrium<br>◯ Left Ventricle<br>◯ Interventricular Septum<br>◯ Tricuspid Valve<br>◯ Mitral Valve<br>◯ Interatrial Septum

25) Estimate the LV systolic function in this patient who has a complicated parapneumonic effusion based on the videos below.
(Please refer to the videos below.)

◯ Hyperdynamic
◯ Normal
◯ Mildly reduced
◯ Moderately reduced
◯ Severely reduced

Match each structure with the appropriate letter in the ultrasound image of the neck below.

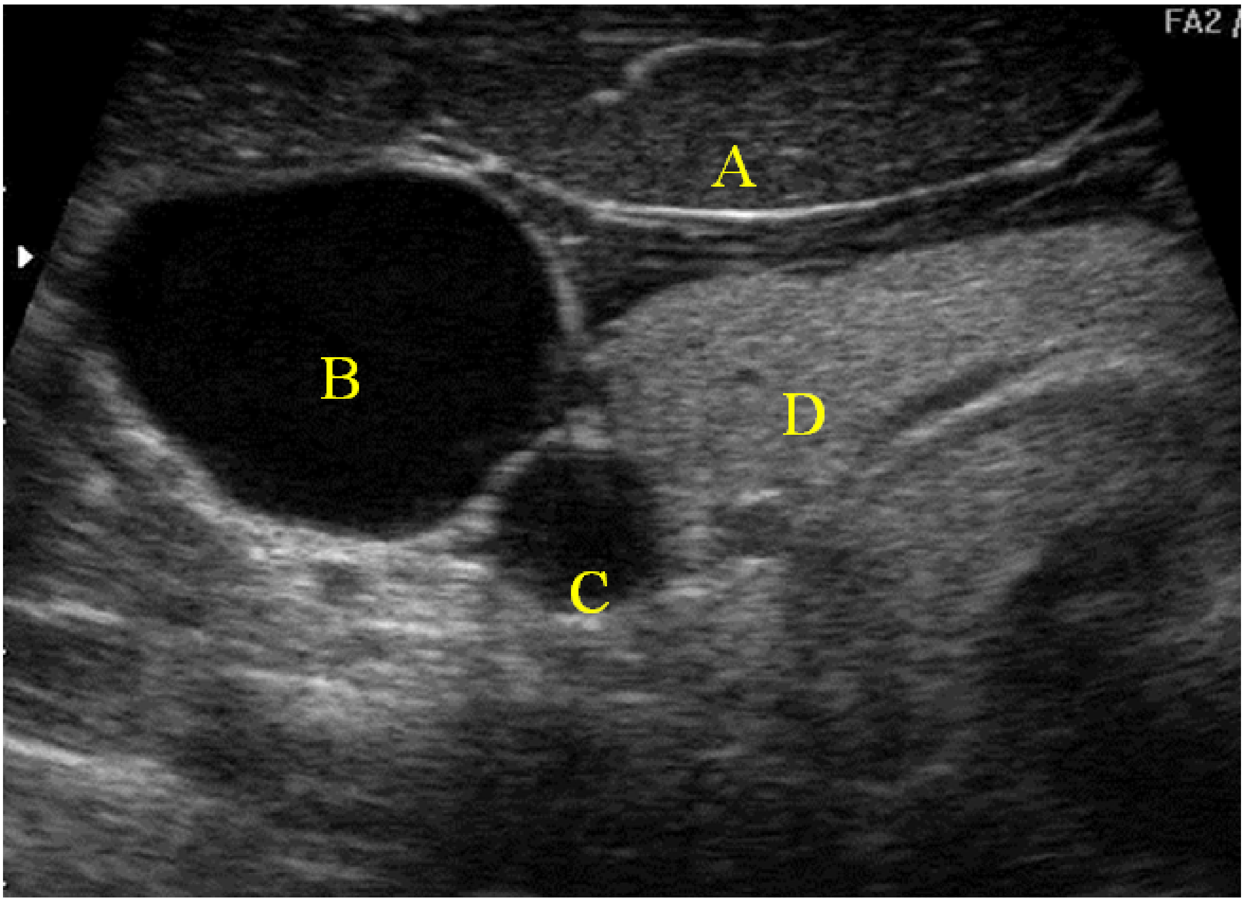

26) Common Carotid Artery
(Please refer to image above.)

◯ A
◯ B
◯ C
◯ D

27) Thyroid Gland
(Please refer to image above.)

◯ A
◯ B
◯ C
◯ D

28) This parasternal long-axis view of the heart shows ________________.
(Please refer to the image below.)

◯ Thoracic aortic aneurysm
◯ Dilated right ventricle
◯ Right pleural effusion
◯ Ascites
◯ Pericardial effusion

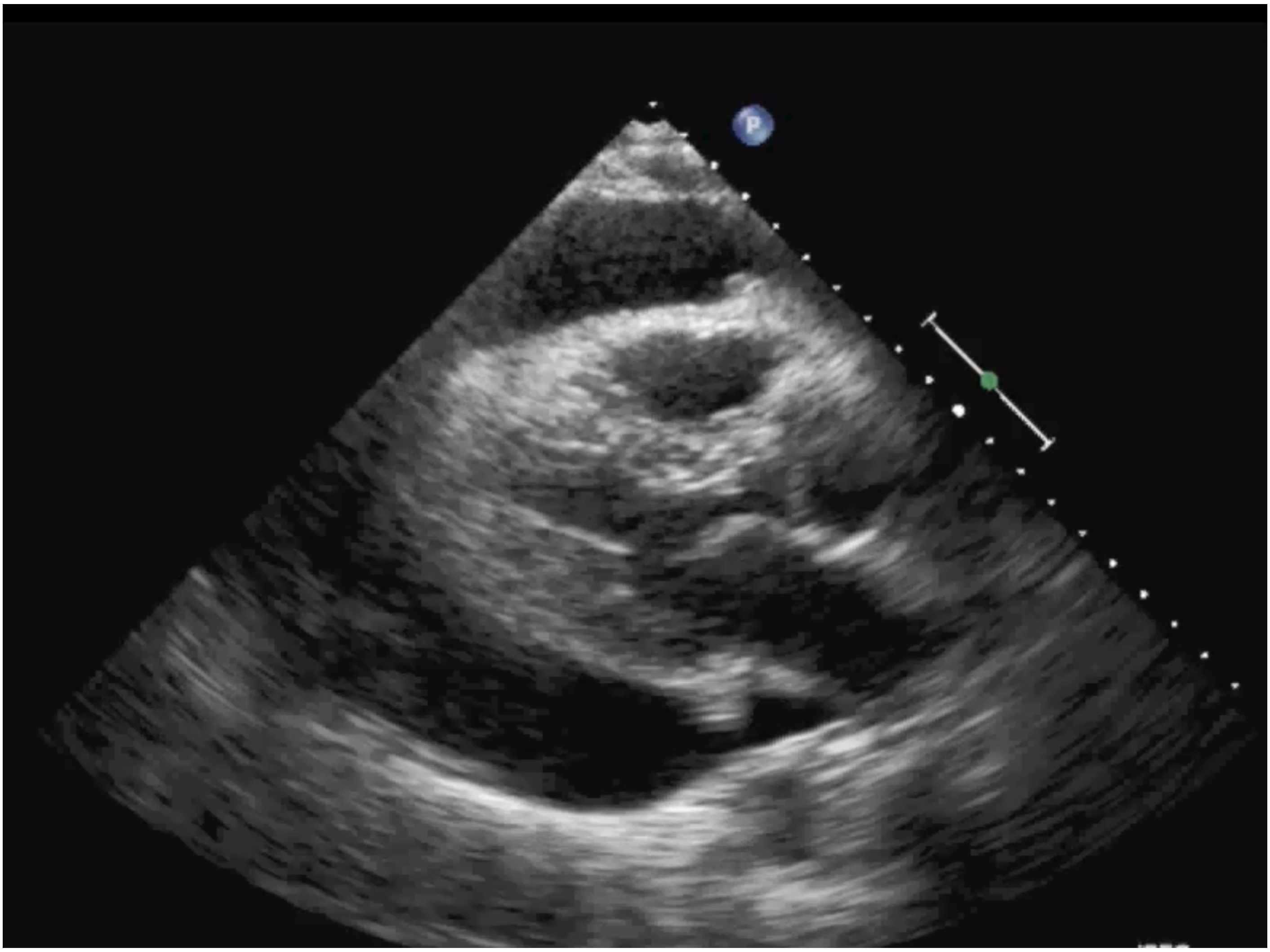

29) What does the image below demonstrate?
(Please refer to the image below.)

◯ Pneumothorax
◯ Pulmonary edema
◯ Pneumonia
◯ Normal lung sliding

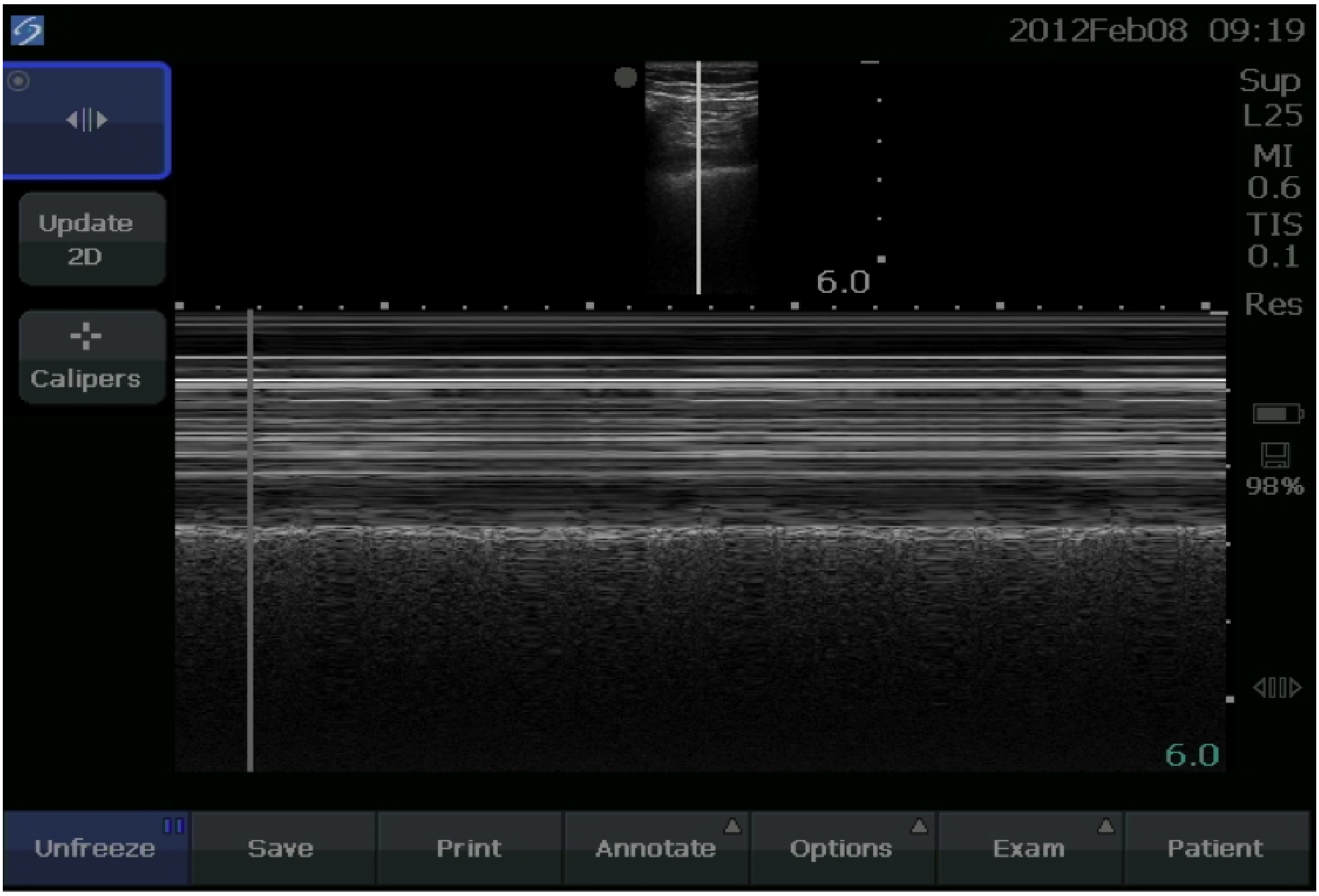

30) What does the image below demonstrate?
(Please refer to the image below.)

◯ Lung mass
◯ Pleural mass
◯ Pleural effusion
◯ Pericardial effusion

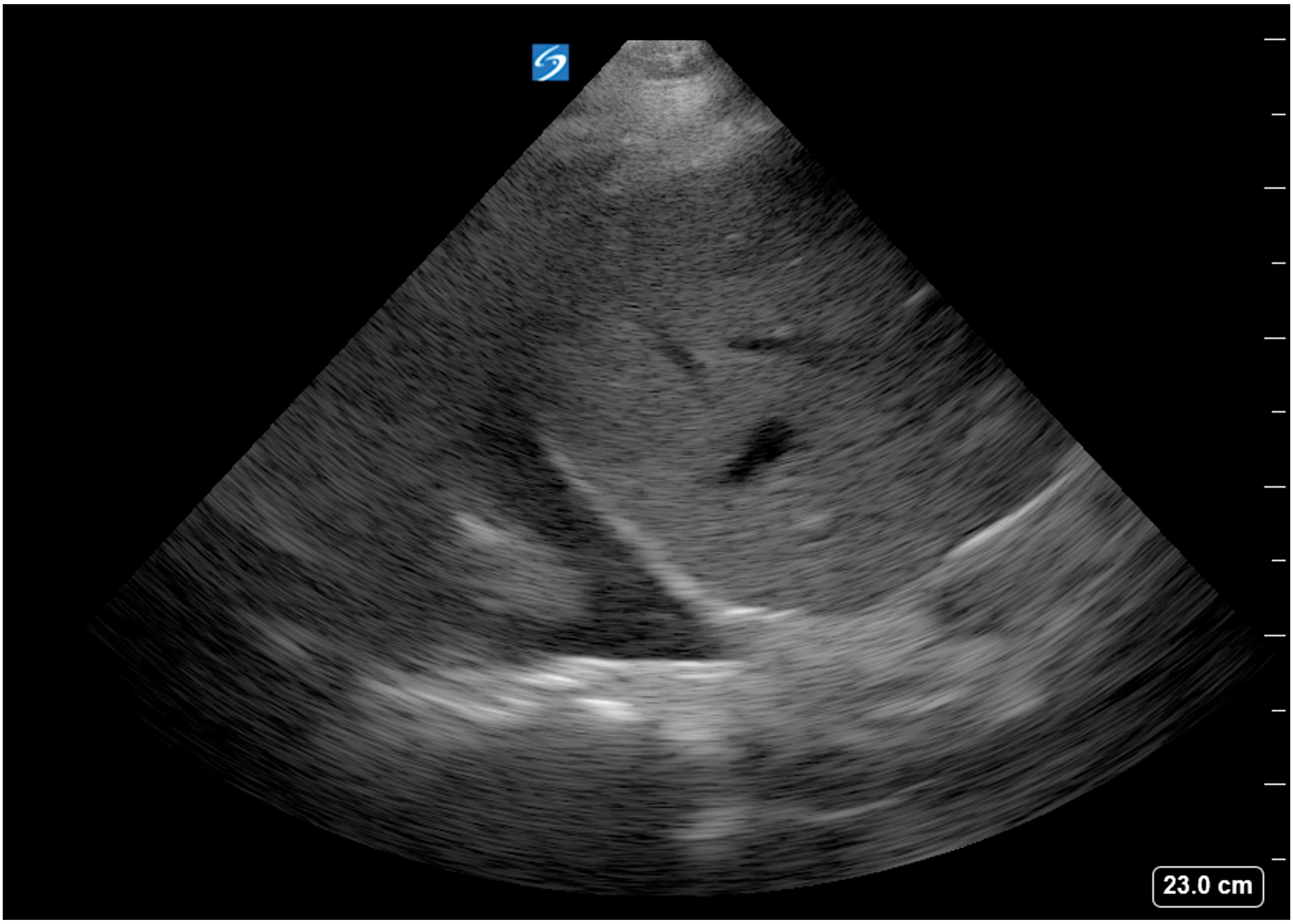

TOTAL SCORE:

(100 max points)

Supplemental File 6. CME Course Evaluation

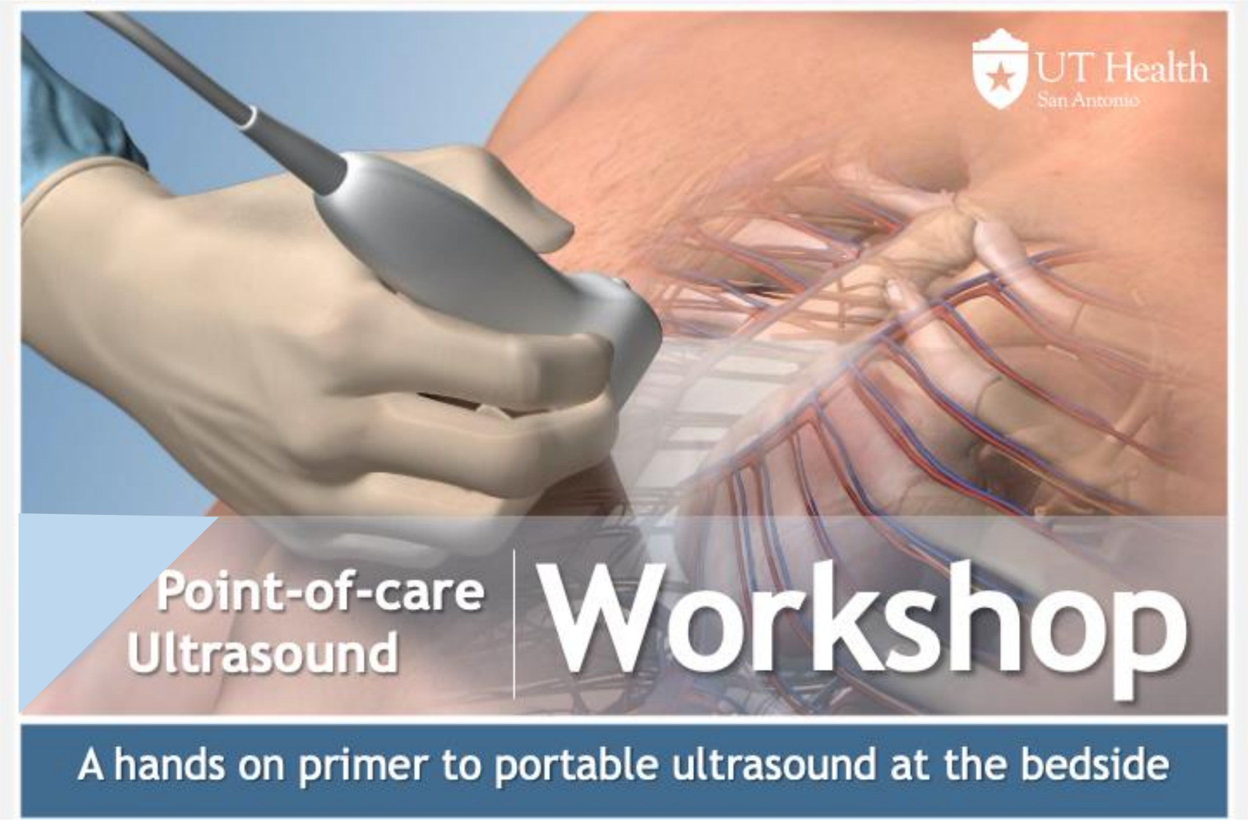

Evaluation Survey

Please complete this evaluation questionnaire. Your anonymous responses will be used to improve this activity and to plan future educational activities.

1. Please select credentials:

- [ ] MD
- [ ] DO
- [ ] PA
- [ ] RN
- [ ] Other

Other (please specify)

Rate the following learning outcomes/objectives:

2. Fundamental principles of ultrasound technology and basic operation of a portable ultrasound machine.

	Not at All	Minimal	Neutral	Good	Excellent
Please select only one.	○	○	○	○	○

3. Techniques to perform focused diagnostic ultrasound examinations at the bedside, including imaging of the heart, lungs, abdomen, and lower extremity veins.

	Not at All	Minimal	Neutral	Good	Excellent
Please select only one.	○	○	○	○	○

4. Techniques to guide performance of bedside procedures with ultrasound guidance, including central venous catheterization, thoracentesis, paracentesis, and lumbar puncture.

	Not at All	Minimal	Neutral	Good	Excellent
Please select only one.	○	○	○	○	○

5. Indications, basic protocols, and limitations of bedside ultrasound imaging.

	Not at All	Minimal	Neutral	Good	Excellent
Please select only one.	○	○	○	○	○

6. Practice interpretation of normal and abnormal ultrasound images.

	Not at All	Minimal	Neutral	Good	Excellent
Please select only one.	◯	◯	◯	◯	◯

7. Mentored scanning with experienced faculty to learn hands-on imaging techniques.

	Not at All	Minimal	Neutral	Good	Excellent
Please select only one.	◯	◯	◯	◯	◯

Part 1 - June 8 - June 21, 2021

Please evaluate speakers.

8. Nilam J. Soni, MD, gave an effective presentation on Getting Started: Ultrasound Equipment & Knobology.

	Not at All	Minimal	Neutral	Good	Excellent
Please select only one.	◯	◯	◯	◯	◯

9. Nilam J. Soni, MD, gave an effective presentation on Focused Cardiac Ultrasound Lecture.

	Not at All	Minimal	Neutral	Good	Excellent
Please select only one.	◯	◯	◯	◯	◯

10. Nilam J. Soni, MD gave an effective presentation on Lung & Plural Ultrasound Lecture.

	Not at All	Minimal	Neutral	Good	Excellent
Please select only one.	◯	◯	◯	◯	◯

11. Nilam J. Soni, MD gave an effective presentation on DVT and Basic Vascular Ultrasound Didactic.

	Not at All	Minimal	Neutral	Good	Excellent
Please select only one.	◯	◯	◯	◯	◯

12. Nilam J. Soni, MD gave an effective presentation on Abdominal Ultrasound Lecture.

	Not at All	Minimal	Neutral	Good	Excellent
Please select only one.	◯	◯	◯	◯	◯

13. Greg Mints, MD gave an effective presentation on Practice Interpretation of Cardiac, Lung and DVT Images.

	Not at All	Minimal	Neutral	Good	Excellent
Please select only one.	◯	◯	◯	◯	◯

Part 2 - June 22 - July 5, 2021

Please evaluate speakers.

14. Benji Matthews, MD gave an effective session on Practice Interpretation: Cardiac Images.

	Not at All	Minimal	Neutral	Good	Excellent
Please select only one.	◯	◯	◯	◯	◯

15. Ria Dancel, MD gave an effective session on Procedures: Vascular Access, Paracentesis, Thoracentesis, Lumbar Puncture.

	Not at All	Minimal	Neutral	Good	Excellent
Please select only one.	◯	◯	◯	◯	◯

16. Charles LoPresti, MD gave an effective presentation on Skin, Soft Tissues, & Joints Lecture.

	Not at All	Minimal	Neutral	Good	Excellent
Please select only one.	◯	◯	◯	◯	◯

Part 3 - July 6 - July 19, 2021

Please evaluate speakers.

17. Ricardo Franco-Sadud, MD gave an effective presentation on Practice Interpretation: POCUS Cases.

	Not at All	Minimal	Neutral	Good	Excellent
Please select only one.	◯	◯	◯	◯	◯

18. Ria Dancel, MD gave an effective presentation on Practice Interpretation: POCUS Cases.

	Not at All	Minimal	Neutral	Good	Excellent
Please select only one.	◯	◯	◯	◯	◯

19. Philip Andrus, MD gave an effective presentation on Cardiac Arrest Lecture.

	Not at All	Minimal	Neutral	Good	Excellent
Please select only one.	◯	◯	◯	◯	◯

Part 4 - July 20 - August 2, 2021

Please evaluate speakers.

20. Nilam J. Soni, MD gave an effective presentation on Ultrasound Program Development.

	Not at All	Minimal	Neutral	Good	Excellent
Please select only one.	◯	◯	◯	◯	◯

21. Benji Matthews, MD gave an effective presentation on Ultrasound Program Development.

	Not at All	Minimal	Neutral	Good	Excellent
Please select only one.	◯	◯	◯	◯	◯

Professional Changes

22. Describe the teaching effectiveness of the faculty overseeing the virtual hands-on sessions.

23. Did the faculty address all of your questions/concerns during the virtual hands-on sessions?

24. Was the Point-of-Care Ultrasound Workshop educational? If so, what new knowledge and/or practice strategies did you gain today?

25. What professional changes will you make to improve your practice and/or care for the patient as a result of attending this workshop? If none, please indicate why.

26. Was this ultrasound workshop evidence-based?

27. Are there other areas of ultrasound you would like addressed?

* 28. Did you find the information to be free of commercial bias, objective, and balanced?

- Yes
- No

Other (please specify)

Overall Accessibility to the Conference

29. Accessibility to the Virtual meeting (lectures and hands-on sessions).

	Not at All	Minimal	Neutral	Good	Excellent
Please select only one.	○	○	○	○	○

30. How did your experience with this year's virtual format impact your learning from being in an in-person workshop?

Comments

* 31. How did you hear about the meeting? Check all that apply.

- [ ] Website
- [ ] Personal recommendation
- [ ] Past Attendee
- [ ] Mailer
- [ ] Email
- [ ] Other

32. Comments?

Supplemental File 7. Tele-ultrasound Course Evaluation

Please complete this course evaluation to help us better understand the effectiveness of virtual point-of-care ultrasound training and improve courses for the future. All responses will be kept confidential and reported in aggregate. Your name and email address will NOT be shared and will only be used for tracking completion of this course evaluation.

Contact Information

First name: ______________________________

Last name: ______________________________

Email address: ______________________________

What is your specialty? (Please select all that apply)

- ☐ Internal Medicine-Primary Care
- ☐ Internal Medicine-Hospitalist
- ☐ Family Medicine
- ☐ Emergency Medicine
- ☐ Critical Care Medicine
- ☐ Pulmonary Medicine
- ☐ Pediatrics
- ☐ Anesthesiology
- ☐ Cardiac Surgery
- ☐ Cardiology
- ☐ General Surgery
- ☐ Thoracic Surgery
- ☐ Vascular Surgery
- ☐ Other

(Mark all that apply)

If you selected other, please specify: ______________________________

Please indicate your role in the course:

- ◯ I was a learner/student enrolled in the virtual course
- ◯ I was only a faculty lecturer
- ◯ I was only faculty for hands-on scanning sessions
- ◯ I was both a faculty lecturer and faculty for the hands-on scanning sessions

Number of years of experience in USING point-of-care ultrasound?

- ◯ < 1
- ◯ 1
- ◯ 2
- ◯ 3
- ◯ 4
- ◯ 5
- ◯ 6
- ◯ 7
- ◯ 8
- ◯ 9
- ◯ 10
- ◯ >10

Have you previously participated in a point-of-care ultrasound CME course that offered hands-on training?

- ◯ Yes
- ◯ No

Please provide details (name of course, when attended, course duration).

____

Number of years of experience in TEACHING point-of-care ultrasound?

- ◯ 1
- ◯ 2
- ◯ 3
- ◯ 4
- ◯ 5
- ◯ 6
- ◯ 7
- ◯ 8
- ◯ 9
- ◯ 10
- ◯ >10

Number of years of experience in USING point-of-care ultrasound?

- ◯ 1
- ◯ 2
- ◯ 3
- ◯ 4
- ◯ 5
- ◯ 6
- ◯ 7
- ◯ 8
- ◯ 9
- ◯ 10
- ◯ >10

VIRTUAL LECTURES

Please answer the following questions as you think about the virtual lectures. The virtual lectures were delivered through Zoom. In addition, lectures were recorded and made available for review at a later time.

How much experience did you have using Zoom for lectures prior to the start of this course?

- ◯ None
- ◯ Some (3-5 lectures)
- ◯ A lot (>5 lectures in past)

Did you have any technical problems with Zoom to view lectures during this course?

- ◯ Yes
- ◯ No

If yes, please describe.

____

Did you watch any of the recorded lectures?

- ◯ Yes
- ◯ No

Rate ease of access to the recorded lectures:

- ◯ Very difficult
- ◯ Somewhat difficult
- ◯ Neutral
- ◯ Somewhat easy
- ◯ Very easy

How effective would you rate the virtual lectures?	◯ Not at all effective<br>◯ Minimally effective<br>◯ Somewhat effective<br>◯ Quite a bit effective<br>◯ Very effective
How would you compare the virtual online lecture format compared to traditional in-person lectures?	__________________________________
Were there any particular lecturing styles that you found were effective?	◯ Yes<br>◯ No
If yes, please describe the lecturing styles you found were more effective:	__________________________________
Were there any particular lecturing styles that you found were less effective?	◯ Yes<br>◯ No
Please describe the lecturing styles you found were not effective:	__________________________________
Were there any particular faculty you found were exceptionally effective for the virtual lectures? Select all that apply.	☐ Phil Andrus<br>☐ Ria Dancel<br>☐ Ricardo Franco<br>☐ Benji Mathews<br>☐ Greg Mints<br>☐ Nilam Soni<br>☐ Other<br>☐ None of the above
What did you find Phil Andrus did effectively?	____________________________
What did you find Ria Dancel did effectively?	____________________________
What did you find Ricardo Franco did effectively?	____________________________
What did you find Benji Mathews did effectively?	____________________________
What did you find Greg Mints did effectively?	____________________________
What did you find Nilam Soni did effectively?	____________________________
Please specify name of other faculty:	____________________________
What did you find this faculty member did effectively?	____________________________

Were there any particular faculty you found were not effective for the virtual lectures? Select all that apply.

- ☐ Phil Andrus
- ☐ Ria Dancel
- ☐ Ricardo Franco
- ☐ Benji Mathews
- ☐ Greg Mints
- ☐ Nilam Soni
- ☐ Other
- ☐ None of the above

What did you find Phil Andrus did less effectively? ______________________________

What did you find Ria Dancel did less effectively? ______________________________

What did you find Ricardo Franco did less effectively? ______________________________

What did you find Benji Mathews did less effectively? ______________________________

What did you find Greg Mints did less effectively? ______________________________

What did you find Nilam Soni did less effectively? ______________________________

Please specify name of other faculty: ______________________________

What did you find this faculty member did less effectively? ______________________________

VIRTUAL SCANNING SESSIONS - SETUP

Please answer the following questions as you think about the setup for the learner-faculty ultrasound scanning sessions that were completed throughout this virtual course.

For your hands-on scanning sessions, who served as your model?

- ☐ Fellow physician or NP/PA
- ☐ Hospital/clinic staff
- ☐ Patient
- ☐ Family member
- ☐ Neighbor or Friend
- ☐ Paid model
- ☐ Other

(select all that apply)

Please specify other: ______________________________

How difficult was it for you to secure a model for each hands-on scanning session?

- ◯ Very easy
- ◯ Somewhat easy
- ◯ Neutral
- ◯ Somewhat difficult
- ◯ Very difficult

How would you rate the ease of setting up the ultrasound machine and computer for the virtual scanning sessions?	◯ Very easy<br>◯ Somewhat easy<br>◯ Neutral<br>◯ Somewhat difficult<br>◯ Very difficult
How would you rate the effectiveness of the teleultrasound software used?	◯ Not at all effective<br>◯ Minimally effective<br>◯ Somewhat effective<br>◯ Quite a bit effective<br>◯ Very effective
The REACTS webapp was the teleultrasound software used primarily during this virtual course. Do you have experience using other types or brands of teleultrasound software?	◯ Yes<br>◯ No
Please select types or brands of teleultrasound software you have experience using:	☐ Lumify with REACTS<br>☐ Butterfly<br>☐ TeamViewer<br>☐ Obs Studio<br>☐ Other<br>(Mark all that apply)
Please specify other:	__________________________________
How would you compare the overall experience with REACTS webapp versus Lumify with REACTS? REACTS webapp was:	◯ Much better<br>◯ Somewhat better<br>◯ Similar<br>◯ Somewhat worse<br>◯ Much worse
How would you compare the overall experience with REACTS webapp versus Butterfly? REACTS webapp was:	◯ Much better<br>◯ Somewhat better<br>◯ Similar<br>◯ Somewhat worse<br>◯ Much worse
How would you compare the overall experience with REACTS webapp versus TeamViewer? REACTS webapp was:	◯ Much better<br>◯ Somewhat better<br>◯ Similar<br>◯ Somewhat worse<br>◯ Much worse
How would you compare the overall experience with REACTS webapp versus Obs Studio? REACTS webapp was:	◯ Much better<br>◯ Somewhat better<br>◯ Similar<br>◯ Somewhat worse<br>◯ Much worse
How would you compare the overall experience with REACTS webapp versus this software? REACTS webapp was:	◯ Much better<br>◯ Somewhat better<br>◯ Similar<br>◯ Somewhat worse<br>◯ Much worse

Please indicate your level of agreement with the statements below

	Strongly disagree	Somewhat disagree	Neutral	Somewhat agree	Strongly agree
The technological setup for the virtual hands on sessions worked better than I had anticipated.	◯	◯	◯	◯	◯
At the beginning of the course, I felt comfortable participating in a virtual hands-on scanning sessions.	◯	◯	◯	◯	◯
By the end of the course, I felt comfortable participating in a virtual hands-on scanning sessions.	◯	◯	◯	◯	◯

Did you encounter any technical problems using REACTS?

◯ Yes
◯ No

Please summarize briefly the technical problems you encountered.

____

Based on your experience, what is the optimal duration of a virtual hands-on scanning session?

◯ 30 minutes
◯ 45 minutes
◯ 1 hour
◯ 1 hour and 15 minutes
◯ 1 hour and 30 minutes
◯ 1 hour and 45 minutes
◯ 2 hours

Any additional comments about the virtual scanning session setup?

____

VIRTUAL SCANNING SESSIONS - FACULTY

Please answer the following questions as you think about the various faculty that led the virtual scanning sessions.

Of the faculty that you worked with, was there anyone who was exceptionally excellent at hands-on teaching that you would recommend serve as faculty for future courses? Select all that apply.

- ☐ Phil Andrus
- ☐ Brandon Boesch
- ☐ Jeremy Boyd
- ☐ Todd Cutler
- ☐ Christopher Dayton
- ☐ Ricardo Franco
- ☐ Elaine Gee
- ☐ Trevor Jensen
- ☐ Gordon Johnson
- ☐ Linda Kurian
- ☐ Gigi Liu
- ☐ Charles LoPresti
- ☐ Benji Mathews
- ☐ Greg Mints
- ☐ Robert Nathanson
- ☐ Kevin Proud
- ☐ Dana Resop
- ☐ Paula Roy-Burman
- ☐ Joseph Ryan James
- ☐ Harold Sauthoff
- ☐ Christopher Schott
- ☐ Jessica Solis-McCarthy
- ☐ David Tierney
- ☐ Jason Williams
- ☐ Tanping Wong
- ☐ Other

What did you find Phil Andrus did effectively? ______________________

What did you find Brandon Boesch did effectively? ______________________

What did you find Jeremy Boyd did effectively? ______________________

What did you find Todd Cutler did effectively? ______________________

What did you find Christopher Dayton did effectively? ______________________

What did you find Ricardo Franco did effectively? ______________________

What did you find Elaine Gee did effectively? ______________________

What did you find Trevor Jensen did effectively? ______________________

What did you find Gordon Johnson did effectively? ______________________

What did you find Linda Kurian did effectively? ______________________

What did you find Gigi Liu did effectively? ______________________

What did you find Charles LoPresti did effectively? __________________________________

What did you find Benji Mathews did effectively? __________________________________

What did you find Greg Mints did effectively? __________________________________

What did you find Robert Nathanson did effectively? __________________________________

What did you find Kevin Proud did effectively? __________________________________

What did you find Dana Resop did effectively? __________________________________

What did you find Paula Roy-Burman did effectively? __________________________________

What did you find Joseph Ryan James did effectively? __________________________________

What did you find Harold Sauthoff did effectively? __________________________________

What did you find Christopher Schott did effectively? __________________________________

What did you find Jessica Solis-McCarthy did effectively? __________________________________

What did you find David Tierney did effectively? __________________________________

What did you find Jason Williams did effectively? __________________________________

What did you find Tanping Wong did effectively? __________________________________

Please specify name: __________________________________

What did you find this faculty member did effectively? __________________________________

Of the faculty that you worked with, was there anyone that you would not recommend serve as faculty for future courses? Select all that apply.

- ☐ Phil Andrus
- ☐ Brandon Boesch
- ☐ Jeremy Boyd
- ☐ Todd Cutler
- ☐ Christopher Dayton
- ☐ Ricardo Franco
- ☐ Elaine Gee
- ☐ Trevor Jensen
- ☐ Gordon Johnson
- ☐ Linda Kurian
- ☐ Gigi Liu
- ☐ Charles LoPresti
- ☐ Benji Mathews
- ☐ Greg Mints
- ☐ Robert Nathanson
- ☐ Kevin Proud
- ☐ Dana Resop
- ☐ Paula Roy-Burman
- ☐ Joseph Ryan James
- ☐ Harold Sauthoff
- ☐ Christopher Schott
- ☐ Jessica Solis-McCarthy
- ☐ David Tierney
- ☐ Jason Williams
- ☐ Tanping Wong
- ☐ Other

What did you find Phil Andrus did less effectively? __________________________________

What did you find Brandon Boesch did less effectively? __________________________________

What did you find Jeremy Boyd did less effectively? __________________________________

What did you find Todd Cutler did less effectively? __________________________________

What did you find Christopher Dayton did less effectively? __________________________________

What did you find Ricardo Franco did less effectively? __________________________________

What did you find Elaine Gee did less effectively? __________________________________

What did you find Trevor Jensen did less effectively? __________________________________

What did you find Gordon Johnson did less effectively? __________________________________

What did you find Linda Kurian did less effectively? __________________________________

What did you find Gigi Liu did less effectively? __________________________________

What did you find Charles LoPresti did less effectively? __________________________________

What did you find Benji Mathews did less effectively? __________________________________

What did you find Greg Mints did less effectively? __________________________________

What did you find Robert Nathanson did less effectively? __________________________________

What did you find Kevin Proud did less effectively? __________________________________

What did you find Dana Resop did less effectively? __________________________________

What did you find Paula Roy-Burman did less effectively? __________________________________

What did you find Joseph Ryan James did less effectively? __________________________________

What did you find Harold Sauthoff did less effectively? __________________________________

What did you find Christopher Schott did less effectively? __________________________________

What did you find Jessica Solis-McCarthy did less effectively? __________________________________

What did you find David Tierney did less effectively? __________________________________

What did you find Jason Williams did less effectively? __________________________________

What did you find Tanping Wong did less effectively? __________________________________

Please specify name: __________________________________

What did you find this faculty member did less effectively? __________________________________

Any other comments about the faculty of the virtual scanning sessions? ____

Compared to other live in-person faculty courses that you have taught, how would you rate each of the following items:

Confidence learners obtained the desired skills by the end of the course.	◯ Not at all confident<br>◯ Slightly confident<br>◯ Somewhat confident<br>◯ Fairly confident<br>◯ Completely confident
Personal level of stress and anxiety as faculty leading a virtual scanning session.	◯ Not at all stressed or anxious<br>◯ Slightly stressed or anxious<br>◯ Somewhat stressed or anxious<br>◯ Fairly stressed or anxious<br>◯ Very stressed or anxious
Ease of providing positive feedback to learners.	◯ Very Difficult<br>◯ Difficult<br>◯ Neutral<br>◯ Easy<br>◯ Very Easy
Ease of providing negative feedback to learners.	◯ Very Difficult<br>◯ Difficult<br>◯ Neutral<br>◯ Easy<br>◯ Very Easy
Clarity of images on your computer screen:	◯ Not at all clear<br>◯ Barely clear<br>◯ Somewhat clear<br>◯ Mostly clear<br>◯ Very clear
Ability to assess image quality.	◯ Not at all able<br>◯ Barely able<br>◯ Somewhat able<br>◯ Mostly able<br>◯ Very able
Ability to see learner's hand position.	◯ Not at all able<br>◯ Barely able<br>◯ Somewhat able<br>◯ Mostly able<br>◯ Very able

Compared to a traditional in-person two-day course, how would you compare the skill level of learners at the completion of the course?

◯ The skill level of learners in the virtual format was better than the in-person format
◯ The skill level of learners in the virtual format was worse than the in-person format
◯ There was no difference in the skill level of learners between the virtual and in-person format

Is there anything else that we haven't asked that you think would be important for us to know about the virtual scanning sessions?	__________________________________

VIRTUAL COURSE OBJECTIVES

As compared to the in-person scanning format, how would you rate the effectiveness of fulfilling each of the following course objectives in virtual course format?

	Not at all met	Minimally met	Somewhat met	Mostly met	Completely met
Fundamental principles of ultrasound technology and basic operation of a portable ultrasound machine:	○	○	○	○	○
Techniques to perform focused diagnostic ultrasound examinations at the bedside, including imaging of the heart, lungs, abdomen, and lower extremity veins:	○	○	○	○	○
Techniques to guide performance of bedside procedures with ultrasound guidance, including central venous catheterization, thoracentesis, paracentesis, and lumbar puncture:	○	○	○	○	○
Indications, basic protocols, and limitations of bedside ultrasound imaging:	○	○	○	○	○
Practice interpretation of normal and abnormal ultrasound images:	○	○	○	○	○
Mentored scanning with experienced faculty to learn hands-on imaging techniques:	○	○	○	○	○

What piece of advice would you most like to give to the faculty to improve this course in the future?

____

What piece of advice would you give to future learners who take this course to maximize their learning?

____

VIRTUAL COURSE OBJECTIVES

As compared to the in-person scanning format, how would you rate the effectiveness of fulfilling each of the following course objectives in virtual course format?

	Virtual format was more effective than the in-person format for this objective	Virtual format was less effective than the in-person format for this objective	There was no difference in effectiveness between the virtual and in-person format for this objective

Fundamental principles of ultrasound technology and basic operation of a portable ultrasound machine:	◯	◯	◯
Techniques to perform focused diagnostic ultrasound examinations at the bedside, including imaging of the heart, lungs, abdomen, and lower extremity veins:	◯	◯	◯
Techniques to guide performance of bedside procedures with ultrasound guidance, including central venous catheterization, thoracentesis, paracentesis, and lumbar puncture:	◯	◯	◯
Indications, basic protocols, and limitations of bedside ultrasound imaging:	◯	◯	◯
Practice interpretation of normal and abnormal ultrasound images:	◯	◯	◯
Mentored scanning with experienced faculty to learn hands-on imaging techniques:	◯	◯	◯

COURSE ADMINISTRATION

How would you rate the overall administration of the course?

- ◯ Poor
- ◯ Below Average
- ◯ Average
- ◯ Very Good
- ◯ Excellent

How would you rate the overall scheduling of the hands-on scanning sessions?

- ◯ Poor
- ◯ Below Average
- ◯ Average
- ◯ Very Good
- ◯ Excellent

We used Outlook calendar invitations for lectures and scanning sessions. How effective was this approach for you personally to keep track of lectures and hands-on scanning sessions?

- ◯ Not effective at all
- ◯ Somewhat ineffective
- ◯ Neutral
- ◯ Somewhat effective
- ◯ Very effective

This virtual course was spread out over weeks. How do you feel about the duration of the course?

- ◯ Way too long
- ◯ Somewhat long
- ◯ Perfect for me
- ◯ Somewhat short
- ◯ Too short

I would recommend this course to a friend or colleague.	◯ Strongly disagree<br>◯ Somewhat disagree<br>◯ Neutral<br>◯ Somewhat agree<br>◯ Strongly agree
Overall, did you feel that the virtual course met your expectations?	◯ Yes<br>◯ No<br>◯ Somewhat
Please elaborate:	__________________________________
Based on your experience, would you participate in another virtual point-of-care ultrasound course?	◯ Yes<br>◯ No<br>◯ Maybe
Please explain why you would not take another virtual course:	__________________________________
Do you have any specific feedback to improve the scheduling of lectures or hands-on sessions?	__________________________________
Were there any unanticipated benefits of the virtual course format?	__________________________________
Were there any unanticipated drawbacks of the virtual course format?	__________________________________
What, if any, are ways we could improve the course for future learners?	__________________________________

Once you have completed the evaluation, please press "Submit." Thank you!

Supplemental Table 8. Characteristics of Learners and Faculty

	In-person	Tele-ultrasound	p-value
**Learners**	70	57	
**Male** *n (%)*	44 (62.9)	29 (51.8)	0.28
**Age** (years) *mean (SD)*	41 (11.3)	37.5 (6.8)	0.038
**Years in Practice** *mean (SD)*	10.3 (10.3)	7.5 (5.0)	0.051
**Degree** *n (%)*			
MD/DO<br>NP/PA<br>Fellow/Resident/Student	55 (78.6)<br>4 (5.7)<br>11 (15.7)	51 (91.1)<br>5 (8.9)<br>0 (0.0)	0.003
**Specialty** *n (%)*			
Internal/Hospital Medicine<br>Family Medicine<br>Critical Care<br>Emergency Medicine<br>Other	41 (58.6)<br>11 (15.7)<br>8 (11.4)<br>2 (2.9)<br>8 (11.4)	34 (60.7)<br>8 (14.3)<br>3 (5.4)<br>2 (3.6)<br>9 (16.1)	0.76
**Frequency of POCUS Use** *n (%)*			
Do not use<br>Few times per year / monthly<br>Few times per week / daily	17 (24.3)<br>35 (50.0)<br>18 (25.7)	13 (23.2)<br>35 (62.5)<br>8 (14.3)	0.24
**Comfort with POCUS** *n (%)*			
Novice<br>Some experience<br>Experienced<br>Proficient	23 (32.9)<br>31 (44.3)<br>14 (20.0)<br>2 (2.9)	19 (33.9)<br>30 (53.6)<br>7 (12.5)<br>0 (0.0)	0.43
**Faculty**	23	26	
**Male** *n (%)*	15 (65.2)	18 (69.2)	1.00
**Age** (years) *mean (SD)*	40 (6.0)	41.4 (9.7)	0.59
**Specialty** *n (%)*			
Internal Medicine<br>Critical Care Medicine<br>Emergency Medicine<br>Other	10 (43.5)<br>5 (21.7)<br>5 (21.7)<br>3 (13.0)	17 (65.3)<br>4 (15.4)<br>5 (19.2)<br>0 (0.0)	0.22
**POCUS Experience** *mean (SD)*			
Clinical Use (years)	9.8 (4.6)	9.3 (4.0)	0.69
Teaching (years)	7.3 (3.9)	7.0 (3.3)	0.74

For continuous variables reported in years, the t-test of 2 independent means with unequal variances was used to calculate the p-value. For categorical variables, the Chi-Squared test was used when all cells had 5 or more participants; otherwise the Fisher exact test was used to calculate the p-value .

Supplemental Table 9. Tele-ultrasound Course Evaluations by Learners and Faculty

	Learners	Faculty
**Virtual Lectures**		
How effective would you rate the virtual lectures?	4.5	3.7
**Tele-ultrasound Scanning Sessions**		
How would you rate the effectiveness of the teleultrasound software used?	4.3	4.1
The technological set-up for tele-ultrasound in the hands-on sessions worked better than I had anticipated.	4.3	4.1
By the end of the course, I felt comfortable participating in the hands-on scanning sessions using tele-ultrasound.	4.6	4.5
**Course Administration**		
This tele-ultrasound course was spread out over weeks. Do you feel the course duration was appropriate from your perspective?	4.7	4.6
I would recommend this course to a friend or colleague.	4.9	4.4
Overall, did you feel that the tele-ultrasound course met your expectations?	5	4.9
Based on your experience, would you participate in another tele-ultrasound point-of-care ultrasound course?	4.8	5

Supplemental Table 10. Faculty Evaluation of the Tele-ultrasound Course

**Tele-ultrasound Course Feedback**	**Faculty**
Confidence learners obtained the desired skills by the end of the course.	4.0*
Ease of providing positive feedback to learners.	4.0*
Ease of providing negative feedback to learners.	3.3*
Compared to a traditional in-person two-day course, how would you compare the skill level of learners at the completion of the course?	
Similar or better than in-person format	89%
Fundamental principles of ultrasound technology and basic operation of a portable ultrasound machine.	
Similar or more effective than in-person format	87%
Techniques to perform focused diagnostic ultrasound examinations at the bedside, including imaging of the heart, lungs, abdomen, and lower extremity veins.	
Similar or more effective than in-person format	69%
Indications, basic protocols, and limitations of bedside ultrasound imaging.	
Similar or more effective than in-person format	100%
Practice interpretation of normal and abnormal ultrasound images.	
Similar or more effective than in-person format	89%
Mentored scanning with experienced faculty to learn hands-on imaging techniques.	
Similar or more effective than in-person format	60%

*5-point scale (1=low agreement; 5=high agreement)
